# Comparative Transcriptome Analysis Identified Candidate Genes Related to Bailinggu Mushroom Formation and Genetic Markers for Genetic Analyses and Breeding

**DOI:** 10.1038/s41598-017-08049-z

**Published:** 2017-08-24

**Authors:** Yongping Fu, Yueting Dai, Chentao Yang, Peng Wei, Bing Song, Yang Yang, Lei Sun, Zhi-Wu Zhang, Yu Li

**Affiliations:** 1 Engineering Research Center of Chinese Ministry of Education for Edible and Medicinal Fungi, Jilin Agricultural University, Changchun, 130118 Jilin China; 2 Department of Crop and Soil Sciences, Washington State University, Pullman, 99164 Washington USA; 3 BGI-Shenzhen, Shenzhen, 518083 China; 4 China National GeneBank, BGI Shenzheng, Shenzhen, 518120 China; 5 Key Laboratory of Integrated Pest Management on Crop in Northwestern Oasis, Urumqi, 830091 Xinjiang China

## Abstract

Bailinggu (*Pleurotus tuoliensis*) is a major, commercially cultivated mushroom and widely used for nutritional, medicinal, and industrial applications. Yet, the mushroom’s genetic architecture and the molecular mechanisms underlying its formation are largely unknown. Here we performed comparative transcriptomic analysis during Bailinggu’s mycelia, primordia, and fruiting body stages to identify genes regulating fruiting body development and develop EST-SSR markers assessing the genetic value of breeding materials. The stage-specific and differentially expressed unigenes (DEGs) involved in morphogenesis, primary carbohydrate metabolism, cold stimulation and blue-light response were identified using GO and KEGG databases. These unigenes might help Bailinggu adapt to genetic and environmental factors that influence fructification. The most pronounced change in gene expression occurred during the vegetative-to-reproductive transition, suggesting that is most active and key for Bailinggu development. We then developed 26 polymorphic and informative EST-SSR markers to assess the genetic diversity in 82 strains of Bailinggu breeding materials. These EST-SSRs exhibited high transferability in closely related species *P*. *eryngii* var. *ferulae* and var. *eryngii*. Genetic population structure analysis indicated that China’s Bailinggu has low introgression with these two varieties and likely evolved independently. These findings provide new genes, SSR markers, and germplasm to enhance the breeding of commercially cultivated Bailinggu.

## Introduction

*Pleurotus tuoliensis* ^1^ is one of the major, choice edible mushrooms with multi-functional nutrients, medicinal properties, and industrial uses^2^. In nature, this mushroom is found in China (Xinjiang, Urumqi) and Iran^3, 4^. In 1997, *P*. *tuoliensis* was first cultivated in China and given the commercial name Bailinggu^5^. Currently, Bailinggu is widely commercially cultivated in China, Japan, and Korea^2, 5^. A variety of substrates, including sawdust, cornstalks, and cottonseed hulls, can be used to cultivate Bailinggu^6, 7^. During the cultivation cycle, environmental factors such as low temperature, appropriate photoperiod, and adequate fresh air are required to trigger and regulate fruiting^7–11^. Yet, the molecular mechanisms underlying Bailinggu’s utilization of substrates and formation of fruiting bodies are largely unknown.

High-throughput transcriptome sequencing using Illumina RNA sequencing technology is a cutting-edge approach that can dramatically improve the effectiveness of gene discovery in edible mushrooms. For example, studies on *Schizophyllum commune* ^12^, *Cordyceps militaris* ^13^, and *Ganoderma lucidum* ^14^ found genes that regulate mushroom formation and demonstrated some of the molecular mechanisms controlling fruiting body development. Additionally, transcriptome sequencing has been successfully applied to develop expressed sequence tag-simple sequence repeat (EST-SSR) markers, allowing genetic diversity analysis, genetic map construction, and QTL mapping^15–18^. For edible mushrooms, the development of ESR-SSR markers from transcriptome sequencing can complement existing genetic diversity analysis and genetic linkage map, and facilitate genotype identification and molecular breeding^3, 19–24^. Prior to the current study, we only used mycelium-stage samples of Bailinggu to develop a few EST-SSR markers^7^. Therefore, further SSR marker development, using additional growth stage samples, is critically needed to advance genetic research of Bailinggu.

The wild population of Bailinggu is distributed in the northern part of Xinjiang Autonomous Region in China and is the major breeding resource. However, this resource is continuously shrinking^3^. Assessments of Bailinggu’s genetic diversity and population genetic structure, using EST-SSR markers, are not only vital for efficient use of genetic resources in breeding, but also for efficient conservation strategy development. Wild Bailinggu are grown with Ferula plants of the family Apiaceae, and belonged to the *P*. *eryngii* species complex that is comprised of species grown on, or in association with, Apiaceae plants^4^. In this mushroom species complex, *P*. *eryngii* var. *eryngii* (commercial name of Xingbaogu) and *P*. *eryngii* var. *ferulae* (commercial name of Aweigu) are the two varieties most closely related to *P*. *tuoliensis* (Bailinggu)^25, 26^. A limited amount of molecular markers are available for genetic studies of these two varieties. EST-SSR markers have a higher level of transferability across phylogenetically related species in plants and fungi^17, 27, 28^. However, no studies have performed a transferability analysis of EST-SSR markers on the *P*. *eryngii* species complex.

Here, we report the findings of our study that used RNA-seq technology to perform a comparative transcriptome analysis of Bailinggu under four major developmental stages. Our specific objectives were the following: (1) investigate relevant functional genes and signaling pathways involved in mushroom formation, (2) screen and identify EST-SSR markers and examine their transferability in Aweigu and Xingbaogu, and (3) illustrate the genetic diversity and population genetic structure of *P*. *tuoliensis* (Bailinggu) and these two *P*. *eryngii* taxa.

## Results

### Four developmental stages of Bailinggu

In the industrialized cultivation cycle of Bailinggu, the developmental process could be divided into four major stages (Fig. S1): undifferentiated mycelia of the physiological after-ripening stage (Stage I), cold stimulation of Stage I mycelia (Stage II), primordia (Stage III), and fruiting body (Stage IV). The Stages I and II represented vegetative growth development of Bailinggu, whereas Stages III and IV represented reproductive growth development. The transition from vegetative to reproductive growth was seldom occurred without cold stimulation and under continuous darkness. Therefore, mycelium was first grown at 25 °C in cultivation bottles up to the physiological after-ripening stage (Stage I), then cold stimulation of them at −3 °C up to 10 days (Stage II) were required to trigger primordia initiation and achieve higher production. After Stage I and II cultivated in the dark, the Stages III and IV were needed to incubate in blue light with 8 hours/day and 1200 lux. Under these conditions, the morphological changes were observed, which the mycelium turned into primordial (Stage III), and following the pinning fruit body appeared in 8–10 days and the fruit body matured (Stage IV) in 15–20 days.

The undifferentiated mycelia of Stage I and II, primordia (Stage III) and fruiting bodies (Stage IV) were used for RNA-seq, which collected from the same batch of cultivated strain CCMJ1077. For each stage, three cDNA libraries were constructed from 9 samples and used as three biological replicas. In total, 12 libraries we construed for four major development stages in this study.

### Illumina sequencing and reads assembly

**Figure 1:**
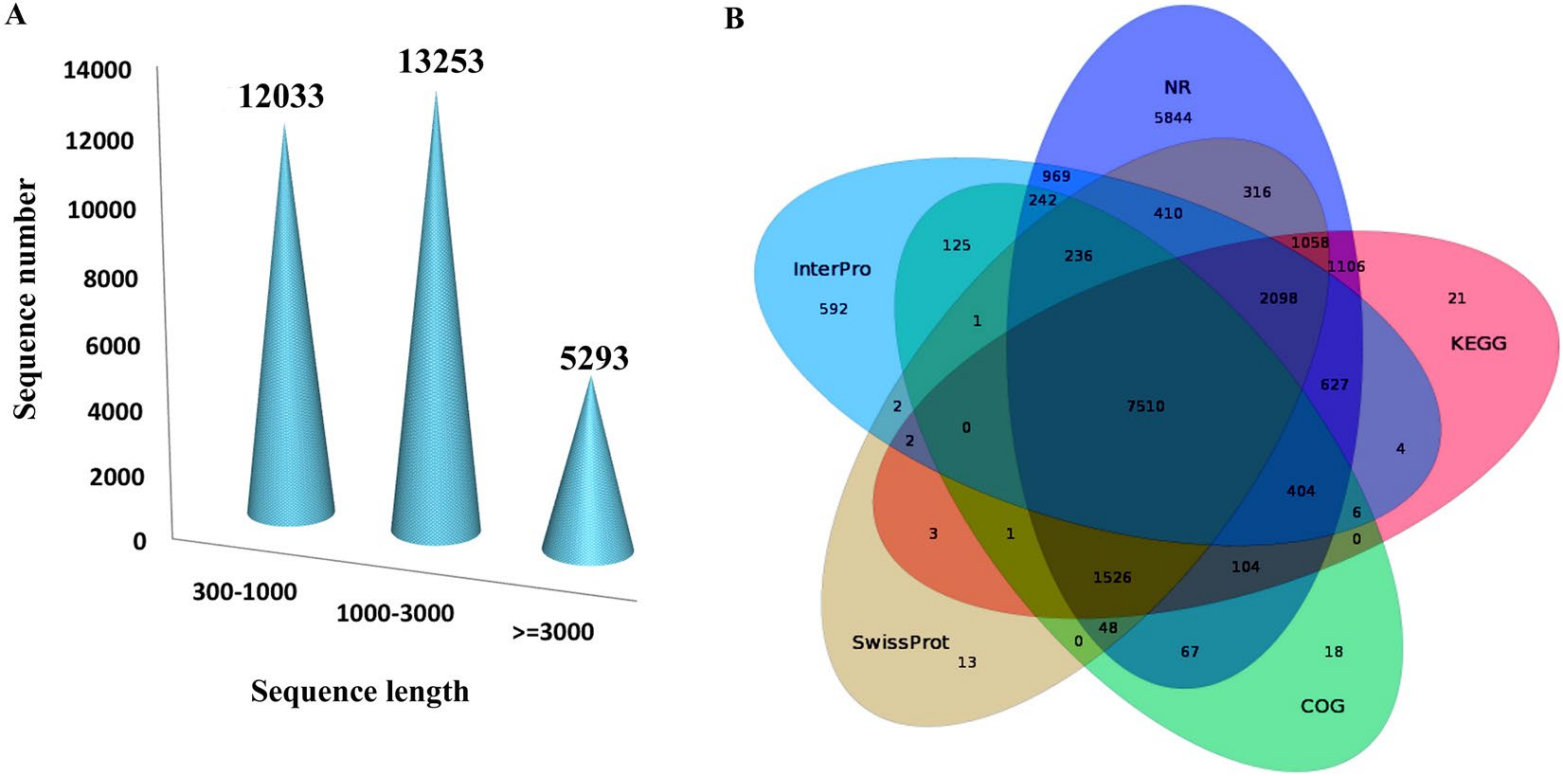
Sequence length distribution and overlap of unigenes based on annotation analyses using five databases. (**A**) A total of 30,578 unigenes were assembled and (**B**) annotated by using five databases: Non-Redundant (NR), SwissProt, InterPro, Clusters of Orthologous Groups of proteins (COG), and Kyoto Encyclopedia of Genes and Genomes (KEGG). Nearly one-fourth of the total number of unigenes were shared by all five databases. However, nearly 20% of unigenes were annotated by only the NR database.

Overall, 198,761,326 raw reads were generated from 12 cDNA libraries of Bailinggu samples representing four major development stages. After data filtering and trimming, 183,284,304 high-quality clean reads were obtained. Then, using Trinity, we *de novo* assembled 30,579 unigenes, with an average length of 1,347 bp ana1d an N_50_ of 2677 bp (Table 1). Of these unigenes, 12,033 (39.4%) had lengths ranging from 300 to 1,000 bp, 13,253 (43.3%) had lengths ranging from 1,000 to 3,000 bp, and 5,293 (17.3%) had lengths of more than 3,000 bp (Fig. 1A).

**Table 1:** Basic statistics of RNA-seq reads obtained from Illumina HiSeq-2500.

Item	Numbers
Total number of raw reads	198,761,326
Total number of clean reads	183,284,304
Total number of unigenes	30,579
Average unigene length (bp)	1,347
Total number of annotated unigenes	23,800
Total number of predicted CDS	22,952
GC content (%) of predicted CDS	52.86

### Functional annotation of the transcriptome

In total, 23,800 unigenes (77.83%) of Bailinggu were annotated and functionally classified (Fig. 1B) using seven public datasets. Among these unigenes, 22,565 (73.79%) matched known proteins in the Non-Redundant (NR) protein database, followed by lower matched percentages in the Kyoto Encyclopedia of Genes and Genomes (KEGG) database (47.32%), Interproscan database (43.26%), Swissprot database (43.25%), Clusters of Orthologous Groups (KOG) database (33.64%), NT database within NCBI (31.17%), and Gene Ontology (GO) database (12.31%). Species distribution analysis showed that 79.83% of these annotated unigenes were homologous with *P*. *ostreatus*, illustrating the closely related interspecific relationship between these two species in the same genus. Furthermore, using Blast and ESTScan, we identified 22,952 of the *de novo*-assembled 30,579 unigenes that had coding sequences (CDS) with an average length of 1,001 bp and an N_50_ of 1,431 bp (Table 1).

We next used the genome sequences of *P*. *eryngii* and *P*. *ostreatus* as the reference genomes to annotate these unigenes and found 22,673 unigenes were annotated. Among them, 21,833 annotated unigenes were same as using NR database, and 19,949 annotated unigenes were belonged to 6,766 genes of *P*. *eryngii*. The rest unigenes should be the different and unique genes in *P*. *tuoliensis*.

The KOG database assigned the matched unigenes to 25 classifications. The largest KOG category was associated with carbohydrate transport and metabolism (1,590 unigenes, 15.5%), followed by amino acid transport and metabolism (1,268, 12.3%); transcription (1,237, 12%); translation, ribosomal structure, and biogenesis (1,110, 10.8%); and replication, recombination, and repair (1,094, 10.6%).

GO analysis revealed 3,764 unigenes related to different GO classes. In the biological process category, the greatest numbers of unigenes were associated with the terms metabolic process (2,027) and cellular process (1,760). In the cellular component category, the greatest numbers of unigenes were associated with the terms cell part (926) and organelle (625). And, in the molecular function category, the greatest numbers of unigenes were associated with the terms binding (1,737) and catalytic activity (2,125). In addition, KEGG results showed that the majority of the 10,288 unigenes were assigned to either metabolism- or genetic information processing-related functional categories.

### Global gene expression analysis

**Figure 2:**
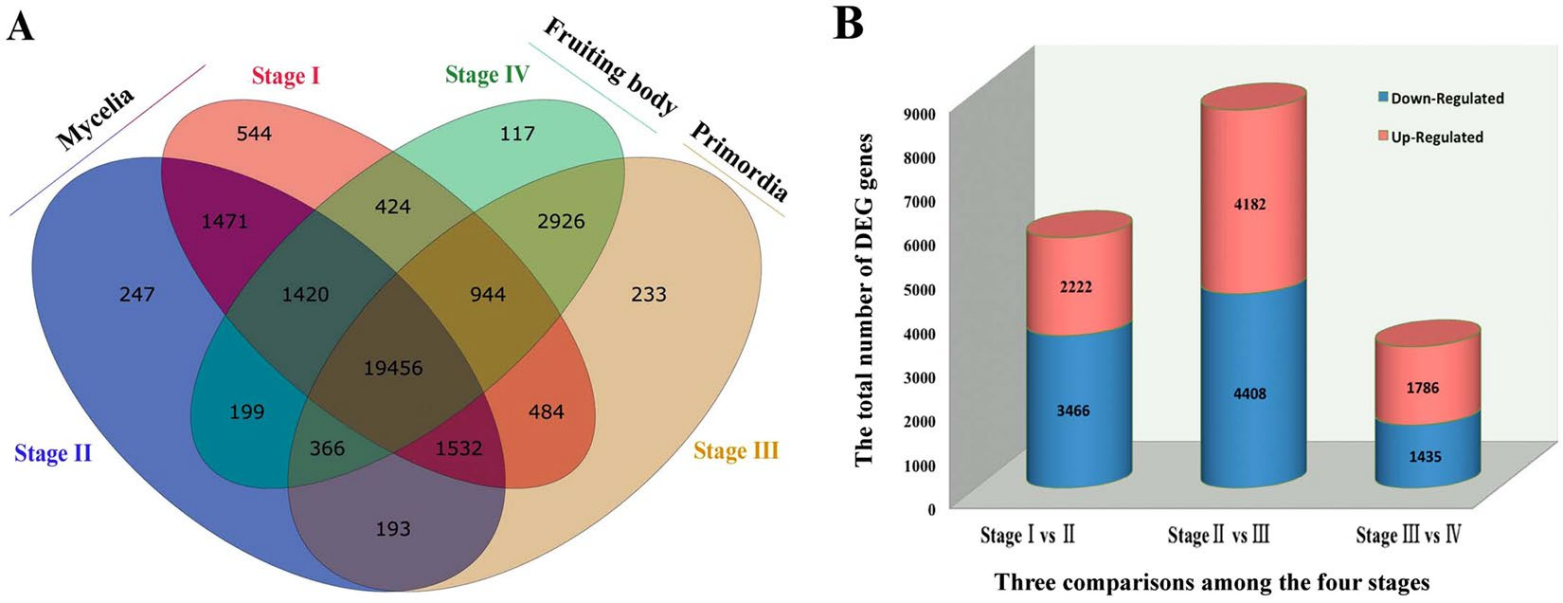
Number of genes expressed or differentially expressed during four developmental stages (tissues) of Bailinggu. Stage I represents physiological after-ripening mycelia, Stage II represents Stage I mycelia plus 10 days of cold stimulation, Stage III represents primordia, and Stage IV represents fruiting body. (**A**) Venn diagram shows unique and common genes expressed across the different developmental stages of Bailinggu transcriptomes. (**B**) Six comparisons among the four stages are displayed along the x-axis. The total number of genes (y-axis) differentially expressed for each comparison are graphically separated into the number that exhibited up-regulated (blue) or down-regulated (red) expression.

To investigate gene expression relationships, we found 19,456 (63.367%) unigenes with shared expressions during all development stages of Bailinggu (Fig. 2A), whereas 544 (1.78%), 247 (0.81%), 233 (0.76%), and 117 (0.38%) genes were specifically expressed only during Stage I, Stage II, Stage III, and Stage IV, respectively. Moreover, the vegetative growth development stage including two mycelia stages (I and II) shared 1,471 stage-specific gene expressions; the reproductive growth development including primordia and fruiting body stages (III and IV) shared 2,926 stage-specific gene expressions.

### The differentially expressed unigenes regulated mushroom formation

To identify and evaluate the differentially expressed unigenes (DEGs), we constructed three DEG libraries to compare Stage I to II, Stage II to III, Stage III to IV. Overall, we detected 2,222, 4,182, and 1,786 up-regulated DEGs and 3,466, 4,408, and 1,435 down-regulated DEGs between Stage I and II libraries, Stage II and III libraries, and Stage III and IV libraries, respectively (Fig. 2B). These results demonstrated that the largest number of DEGs occurred during the vegetative-to-reproductive transition stage—from II to III—suggesting that this time period was the most active and, thus, key for Bailinggu development.

**Figure 3:**
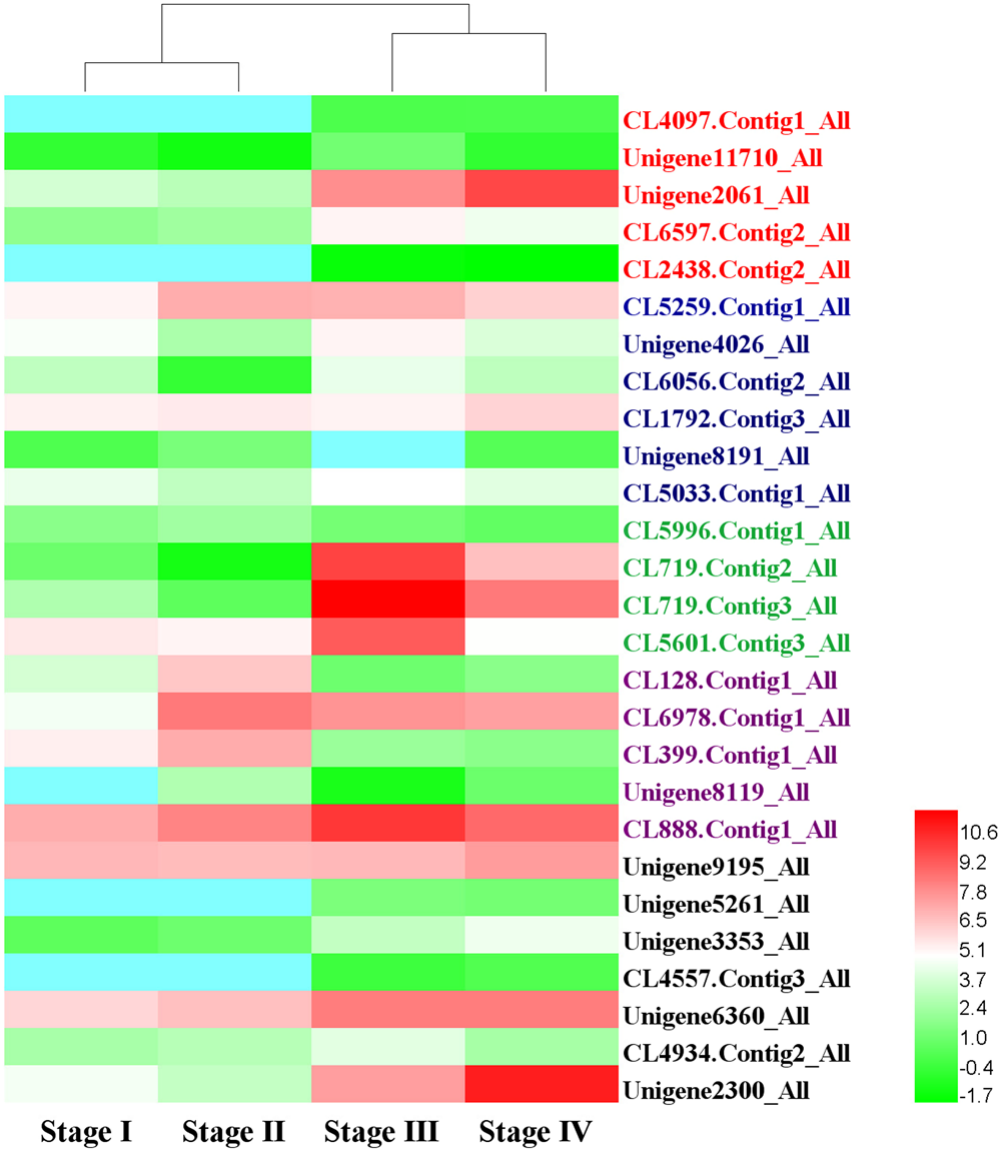
Heat map of differential gene expression associated with fruiting structure formation in Bailinggu. Stage I represents physiological after-ripening mycelia, Stage II represents Stage I mycelia plus 10 days of cold stimulation, Stage III represents primordia, and Stage IV represents fruiting body. These genes include unigenes involved in development and reproduction, and responding to cold and blue light stimulation.

To investigate DEGs involved in development and reproduction, we performed functional enrichment analysis using all DEGs against GO database. Five unigenes associated with GO terms related to developmental process, reproduction and reproductive process were identified, and showed up-regulation during the vegetative-to-reproductive transition, including CL4097.Contig 1_All, Unigene11710_All, Unigene2061_All, CL6597.Contig2_All, and CL2438.Contig2_All (Fig. 3). Among them, the unigenes (Unigene2061_All) directly related to fruiting body development was also up-regulated expression during reproductive growth.

We next examined DEGs belonged to the transcription factor, which found to be essential for growth and reproduction, using the pipeline of transcription factor database and the functionally annotated unigene databases. The MADS-box gene (CL5259.Contig1_All) (Fig. 3) that is a transcription factor of morphogenesis was identified, and exhibited up-regulated expression during the cold stimulation of mycelia, primordia, and fruiting body stages. Moreover, other transcription factors were also identified (Fig. 3), such as the unigenes *c2h2* (Unigene4026_All), *hom* (CL6056.Contig2_All), *fst3* (CL1792.Contig3_All), *fst4* (Unigene8191_All), and *gata* (CL5033.Contig1_All). These unigenes were up-regulated in either the primordia or fruiting body stage, or in both stages.

To further investigate the functional pathways involved in the fruiting process, we utilized all DEGs to match the KEGG database. We found that unigenes involved in the cell cycle (ko04111), meiosis (ko04113), ribosome (ko03010), and mitogen-activated protein kinases (MAPK) signaling (ko04011) pathways were showed up-regulated expression in one or both reproductive growth stages. In the cell cycle and meiosis pathways (Fig. S2), these DEGs included genes encoding cyclin dependent kinase (CDK) (Cdc28, Unigene1829_All), spindle checkpoint (Mps1, Unigene1578_All), DNA damage checkpoint (Rad53, Unigene6136_All), and other cell-cycle-regulating genes. In the ribosome pathway, we found the unigenes encoded 91 ribosomal proteins showed up-regulated expressions when we compared Stage II with Stage III (Fig. S3). It indicated that the ribosome pathway might play a vital role in cell differentiation and development in Bailinggu. Of the MAPK signaling pathways (Fig. S4), we observed up-regulated expression unigenes specifically related to pheromone-dependent and starvation-dependent MAPK signaling pathways during fruiting. These DEGs included unigenes encoding MATα, Ste3, sho1, Ste4, Cdc42, Ste11, and Ste7.

We also identified DEGs involved in the primary carbohydrate metabolism pathways, such as the glycolysis pathway (ko00010) and the tricarboxylic acid (TCA) cycle (ko00020), in the four developmental stages of Bailinggu. We found increased expression of unigenes involved in the glycolysis pathway (Fig. S5). In the energy investment phase of glycolysis, these unigenes encoded phosphoglucomutase (CL5366.Contig1_All), glyceraldehyde 3-phosphate dehydrogenase (CL5201.Contig5_All), and phosphoglycerate kinase (Unigene5744_All). In the energy generation phase, unigenes involved in pyruvate and phosphoenol-pyruvate metabolism, including phophoenolpyruvate carboxylase (CL4166.Contig1_All), enolase (CL5693.Contig1_All), pyruvate kinase (Unigene4245_All), and L-lactate dehydrogenase (Unigene10740_All), also exhibited increased expression in the fruiting body development stage. In addition, the unigenes in the TCA cycle pathway included genes encoding dihydrolipoamide acetyltransferase (Unigene3656_All), fumarate hydratase (Unigene7167_All), citrate synthase (Unigene5611_All), 2-oxoglutarate dehydrogenase (Unigene3667_All), and isocitrate dehydrogenase (Unigene124_All). These unigenes were expressed more abundantly in the reproductive growth stages compared to the vegetative growth stages.

Finally, according to the genes involved in fruit body development of *Pleurotus ostreatus* ^28–32^, we examined them in our three DEG libraries of Bailinggu (*P*. *eryngii tuoliensis*). We found some unigenes in accordance with previous observations, such as unigenes encoded hydrophobin, metalloprotease, cytochrome P450, septin, and glycoside hydrolase family 5 protein. For example, we found 4 unigenes encoding three kind of hydrophobin homologous with *P*. *ostreatus* in four developmental stages (Fig. 3). Among them, the unigene encoding vegetative mycelium hydrophobin 1 (CL5996.Contig1_ALL) showed down-expressed in the reproductive growth stages, whereas unigenes encoding fruit-body hydrophobin 1 (CL719.Contig2_ALL and CL719.Contig3_ALL) were found to be significantly up-expressed in primordia and fruiting body. The unigene encoding hydrophobin 2 (CL5601.Contig3_ALL) was up-expressed in primordia while down-expressed in the fruiting bodies. However, we did not found PriA that specifically expressed during the initiation of fruiting.

### Identification of unigenes involved in cold and light response

To trigger primordia initiation and achieve higher production of Bailinggu, cold stimulation of the cultivation bottles (Stage II) were required after the mycelia grown at 25 °C up to physiological after-ripening stage (Stage I). To identify the genes involved in cold stimulation, we compared the Stage I with Stage II and found the cold-responsive genes. According to GO and KEGG results, these DEGs were mainly related to cell wall and membrane systems, calcium signaling, soluble sugar and protein biosynthesis and metabolism, and antioxidant enzymatic defense system (Fig. 3). For example, we found unigenes that encoded phospholipase (CL128.Contig1_All), fatty acid desaturases (CL6978.Contig1_All), calcium-dependent protein kinase (CL399.Contig1_All), catalase (Unigene8119_All), and heat shock protein (CL888.Contig1_All). Using samples during different temporal stages of cold stimulation, we previously demonstrated that these cold-responsive genes might help Bailinggu adapt to cold temperatures that could trigger fruiting body development^6^.

Among the environmental factors, blue light stimulation is another main trigger for primordia initiation and fruiting body growth of Bailinggu. After the mycelia (Stage I and II) cultured in the dark, the Stage III and IV were needed cultivation under the blue light. To investigate unigenes involved in blue-light response, we compared DEGs between the four different development stage samples. The unigenes involved in blue-light response with up-regulated expression between the mycelia (Stage I and II) and primordia (Stage III) were identified (Fig. 3). However, we found these unigenes lacked differential expression between Stages I and II mycelia. These associated genes included unigenes encoding for white collar-1 (WC-1) (Unigene9195_All), light-regulated protein Lir1 (Unigene5261_All), photolyase (Unigene3353_All), and cryptochrome (CL4557.Contig3_All). We also identified 40 unigenes, including Unigene6360_All, CL4934.Contig2_All, and Unigene2300_All, with up- or down-regulated expression that annotated as being regulated by blue-light stimulation during the four developmental processes.

### Development, characterization, and transferability of EST-SSR markers

To further develop new SSR markers for Bailinggu, we used the MIcroSAtellite (MISA) identification tool to identify all assembled unigenes and found 2,147 potential EST-SSRs from 1,931 unigenes. Of these 1,931 unigenes, 176 contained more than one EST-SSR. We also found 62 EST-SSRs present in compound formation. Of the 2,147 potential EST-SSRs, tri-nucleotide repeats, with a frequency of 50.12%, were the most commonly repeated motif, followed by di-nucleotide repeats (23.33%), mono-nucleotide repeats (10.29%), hexa-nucleotide repeats (8.15%), penta-nucleotide repeats (5.45%), and tetra-nucleotide repeats (2.65%). All above-mentioned data are shown in Table 2. The most dominant di-nucleotide and tri-nucleotide repeat motifs were AG/CT (222, 10.34%) and ACG/CGT (224, 10.43%), respectively.

**Table 2:** Summary statistics of EST-SSRs generated from Bailinggu transcriptome.

Item	Numbers
Total number of unigenes examined	30,579
Total number of identified SSRs	2,147
Number of SSRs containing unigenes	1,931
Number of sequences containing more than one SSR	176
Number of SSRs present in compound formation	62
Number of mono-nucleotide SSRs	10.29%
Number of di-nucleotide SSRs	23.33%
Number of tri-nucleotide SSRs	50.12%
Number of tetra-nucleotide SSRs	2.65%
Number of penta-nucleotide SSRs	5.45%
Number of hexa-nucleotide SSRs	8.15%
SSRs located in the CDS	629
SSRs located in the UTR	739

We investigated the distribution of these potential EST-SSRs and found that 629 and 739 were located in the CDS and untranslated region (UTR), respectively. Of 629 potential EST-SSRs located in the CDS, most (53%) were the tri-nucleotide type. Using Premier 3 software, we then successfully designed 1,693 primer pairs for these EST-SSRs. To verify these potential EST-SSRs, we randomly selected 100 primers for synthesis and amplification (Supplementary Table S1), using 10 wild stains of Bailinggu. We found that four primers failed to amplify any Bailinggu products; 23 and 73 primers amplified monomorphic and polymorphic products, respectively (Supplementary Table S1). The overall amplification rate was 96% and the polymorphism rate was 73%.

We also performed the across-taxa transferability analysis of these 100 EST-SSRs in the two varieties of *P*. *eryngii*, Aweigu and Xingbaogu. In Aweigu, 18 primers failed to amplify any products; 37 and 45 primers amplified monomorphic and polymorphic products, respectively (Supplementary Table S1). In Xingbaogu, 19 primers failed to amplify any product; 36 and 45 primers amplified monomorphic and polymorphic products, respectively (Supplementary Table S1). Therefore, the overall transferability rates were 82% in Aweigu and 81% in Xingbaogu. These polymorphic primers can be used for subsequent genetic diversity analyses of *P*. *eryngii* species complex populations, especially for Bailinggu, Aweigu, and Xingbaogu.

### Genetic diversity, relationship, and population structure analyses using EST-SSRs

We isolated 47 wild strains from wild fruiting bodies growing with Apiaceae plants in the cities of Tahe, Shihezi, Tuoli, Fuyun, and Qinhe located in the Xinjiang Autonomous Region of China. The 47 strains included 35 *P*. *tuoliensis* (Bailinggu) and 12 *P*. *eryngii* var. *ferulae* (Aweigu). We also collected 35 cultivated strains from different factories in China, including 17 Bailinggu, three Aweigu, and 15 *P*. *eryngii* var. *eryngii* (Xingbaogu). Thus, in total, we analyzed 52, 15, and 15 strains of Bailinggu, Aweigu, and Xingbaogu, respectively (Supplementary Table S2). Further, the variation in agricultural characteristics of these 82 strains were observed, such as mushroom shapes ranging from columnar to palmate, cap colors being white to brown, the cultivated cycle ranging from 60 to 120 days. Moreover, the mycelium run rate, days required for primordial initiation, the primordia initiation number, and the yield in these strains also varied significantly.

We selected 26 polymorphic and stable EST-SSR markers (Table 3) to evaluate the genetic diversity of 82 strains of the above *P*. *eryngii* species complex populations. First, 128 alleles were detected across 52 strains of *P*. *tuoliensis* (Bailinggu). The GenAIEx (Ver. 6.502) and CERVE (Ver. 3.0.7) software analyses showed that number of alleles per locus (Na), observed heterozygosity (H_o_), expected heterozygosity (H_e_), Shannon’s information index (I), and polymorphism information content (PIC) values ranged from 1–8 (mean = 4.923), 0.000–0.692 (mean = 0.319), 0.000–0.766 (mean = 0.439), 0.000–1.614 (mean = 0.855), and 0.598–1.905 (mean = 0.396), respectively (Table 3). Moreover, 210 alleles were detected across all 82 strains of the *P*. *eryngii* species complex. For the three *P*. *eryngii* taxa population, values for Na, Ho, He, I, and PIC ranged from 2–13 (mean = 8.077), 0.085–0.683 (mean = 0.681), 0.393–0.843 (mean = 0.674), 0.646–2.135 (mean = 1.430), and 0.339–0.825 (mean = 0.629) (Supplementary Table S3). The genetic diversity parameters for the Xingbaogu and Aweigu populations are detailed in Supplementary Tables S4 and S5, respectively. These results indicated a relatively high level of genetic diversity among the 52 strains of *P*. *tuoliensis* (Bailinggu) and among 82 strains of the *P*. *eryngii* species complex populations. This genotype results closely matched the phenotype results in these 82 different strains.

**Table 3:** Characteristics of the 26 polymorphic EST-SSR markers in the Bailinggu population.

Locus	N	Na	Ne	Ho	He	I	PIC
BlgSSR1	52	4	1.408	0.269	0.293	0.6117	0.275
BlgSSR2	52	4	1.6635	0.365	0.403	0.7857	0.371
BlgSSR3	52	4	1.2627	0.231	0.21	0.4229	0.193
BlgSSR4	52	5	2.04	0.327	0.515	0.955	0.456
BlgSSR5	52	3	1.2412	0.212	0.196	0.4069	0.185
BlgSSR6	52	7	1.6932	0.308	0.413	0.9277	0.394
BlgSSR7	52	6	1.8087	0.269	0.451	0.9669	0.427
BlgSSR8	52	3	1.145	0.019	0.128	0.2742	0.121
BlgSSR9	52	2	1.9817	0.481	0.5	0.6885	0.373
BlgSSR10	52	4	1.8401	0.404	0.461	0.819	0.406
BlgSSR11	52	6	2.3111	0.538	0.573	1.0263	0.484
BlgSSR12	52	5	3.0128	0.423	0.675	1.2632	0.611
BlgSSR13	52	7	4.1504	0.365	0.766	1.5617	0.721
BlgSSR14	52	5	2.1728	0.577	0.545	0.9161	0.447
BlgSSR15	52	7	2.2046	0.385	0.552	0.93	0.443
BlgSSR16	52	8	2.4371	0.615	0.595	1.2682	0.557
BlgSSR17	52	6	1.2719	0.231	0.216	0.5219	0.208
BlgSSR18	52	7	2.2384	0.635	0.559	1.1219	0.509
BlgSSR19	52	1	1	0	0	0	0
BlgSSR20	52	3	1.3726	0.154	0.274	0.5311	0.253
BlgSSR21	52	5	1.986	0.269	0.501	0.9502	0.45
BlgSSR22	52	3	2.9328	0.25	0.665	1.0875	0.585
BlgSSR23	52	8	1.8902	0.346	0.476	1.0616	0.448
BlgSSR24	52	7	3.8464	0.692	0.747	1.6142	0.712
BlgSSR25	52	7	3.1981	0.346	0.694	1.5238	0.663
BlgSSR26	52	1	1	0	0	0	0
Mean	52	4.923	2.043	0.335	0.439	0.855	0.396

**Figure 4:**
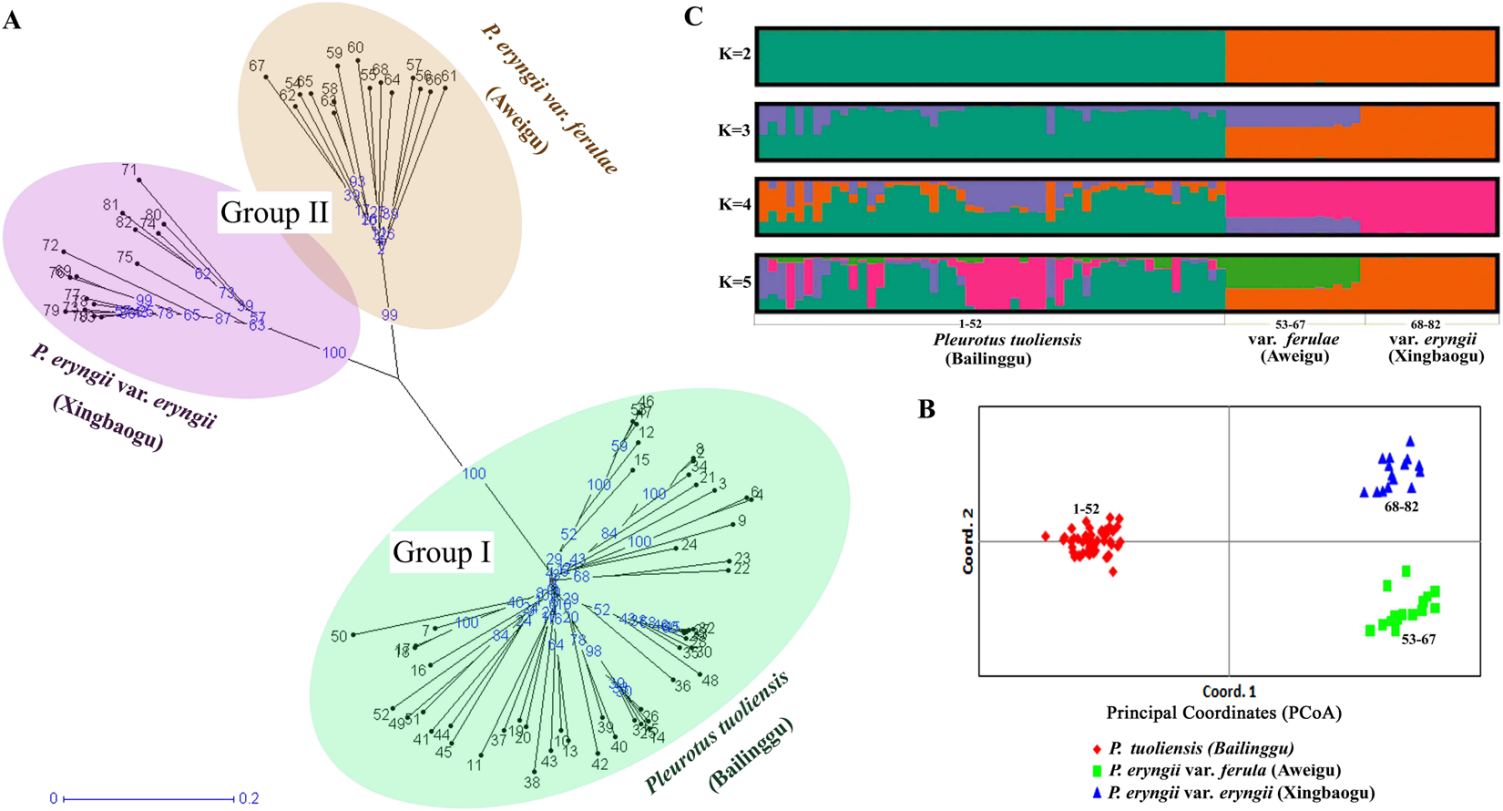
EST-SSR markers reveal population structure and unrooted tree of 82 individuals from three taxa. The three taxa are one species (Bailinggu) and two varieties (Aweigu and Xingbaogu) o *Pleurotus eryngii*, containing 52, 15, and 15 individuals, respectively. These 82 strains include 47 wild and 35 cultivated strains. The variable phenotypes are observed, which shape from columnar to palmate, cap color from white to brown, cultivated cycle from 60 to 120 days. To analysis of genetic diversity and population structure, 26 EST-SSR markers were used to (**A**) conduct the neighbor-joining tree, (**B**) principal coordinate analysis, and (**C**) population structure analysis. Individuals are labeled in black and the distances are labeled in blue on the unrooted tree.

We then investigated the genetic relationships among these *P*. *eryngii* species-complex populations using the above combined profiles of 26 EST-SSR markers. Based on GenAIEx (Ver. 6.502) software analysis results, the Nei’s genetic distance and genetic identity values were 1.796 and 0.166 between Bailinggu and Xingbaogu population, 1.655 and 0.191 between Bailinggu and Aweigu population, and 0.89 and 0.411 between Xingbaogu and Aweigu population. The neighbor-joining (NJ) tree (Fig. 4A) was constructed using Darwin software and showed that all 82 strains were clustered into two distinct groups. Group 1 included all 52 strains of Bailinggu. Group 2 was further divided into two subgroups, one comprised of 15 strains of Aweigu and the other with 15 strains of Xingbaogu. As expected, Bailinggu were separated from the two subgroup variety strains (Aweigu and Xingbaogu). Principal Coordinate Analysis (PCoA) revealed that 55.26%, 15.91%, and 2.86% of the variance was explained by the first, second, and third coordinate axes, respectively (Fig. 4B). Thus, the total explained variation was 74.03%. The PCoA results concurred with the NJ-tree that showed separation of these three populations.

According to the above combined profiles of 26 EST-SSR markers, we finally analyzed the population genetic structure of these 82 strains of the *P*. *eryngii* species complex populations using STRUCTURE software and the STRUCTURE HARVESTER program. Results showed that the likelihood is optimum at K = 2, suggesting that Bailinggu is distinguishable from the two established varieties, Aweigu and Xingbaogu (Fig. 4C). The first inferred genetic group includes all 52 strains of Bailinggu with more than 90% co-ancestry values. The second genetic group is comprised of all 30 strains of the two varieties (Aweigu and Xingbaogu) with more than 90% co-ancestry values. However, the fraction of population structure under K = 3 suggests that Aweigu deviated from Xingbogu (Fig. 4C). Analysis of molecular variance (AMOVA) indicated that the genetic differentiation was 50% for both among and within population comparisons, whereas the variation among and within individuals was 11% and 39%, respectively. Fixation index (Fst), gene flow (Nm) based on Fst value, and genetic differentiation coefficient (Gst) values among the three populations were 0.5, 0.25, and 0.39, respectively.

## Discussion

Following the high-throughput RNA sequencing approach, we conducted a comparative transcriptomic analysis to reveal which genes varied in abundance among four major developmental stages in Bailinggu. We then used this information to identify potential functional group genes responsible for regulating the formation of this culturally valuable mushroom. During the cultivation cycle, cold and light stimulation are essential for vegetative-to-reproductive transition and fruiting body formation^7–11^. Therefore, we performed analyses of gene expression patterns involved in these stimulation periods, in cellular and physiological processes, and in morphological changes during these developmental stages. We found that stage-specific genes and DEGs might help regulate Bailinggu’s adaptation to genetic and environmental factors such as light and cold temperatures that influence fructification.

Transcription factors that regulate mushroom formation in fungi^12, 32–34^ and fertilization in plants^35, 36^ have been identified. In our study, we identified some transcription factors that might play an important role in mushroom formation and cold stress response of Bailinggu, such as MADS, C2H2, and FST4. The MADS genes of *Arabidopsis thaliana* are essential for flowering and help to ensure that fertilization occurs at the time of maximal reproductive potential^35, 36^. In Bailinggu, the MADS gene sustained up-regulated expression during the later three developmental stages, indicating that it may be essential for Bailinggu morphogenesis. Ohm *et al*. (2010) found that the *fst4* gene is up-regulated during fruiting body development in *S*. *commune*. Additionally, they observed that *S*. *commune* failed to fruit when the *fst4* gene was inactivated in dikaryon. Therefore, we suggest that these unigene-encoding transcription factors are important resources for future studies of primordia development and fruiting body formation in Bailinggu.

Previous studies have shown that many KEGG pathways are associated with fruiting in fungi, such as primary carbohydrate metabolism^37, 38^, cell cycle^39^, and MAPK signaling pathways^40, 41^. We found that cells employ the TCA cycle pathway to a greater extent during reproductive growth stages compared to vegetative growth stages, indicating that more energy is requested during reproductive growth. This finding suggests that the TCA cycle pathway might play a crucial role in providing energy and the carbon backbone for compounds necessary for cell growth, proliferation, and development in Bailinggu. Moreover, the major genes encoding the glycolytic enzymes in the glycolysis pathway were up-regulated in fruiting body development, compared to the other three developmental stages of Bailinggu. We speculate that the accelerated rate of glycolysis during the fruiting process could consume most of the accumulated compounds utilized for fruiting body formation.

We observed that unigenes related to pheromone-, starvation-, and osmolarity-dependent MAPK signaling pathways were differentially expressed during all four stages. This finding indicates that unigenes in MAPK signaling pathways might be mainly involved in cell growth and differentiation, mycelia development, fruiting body morphology, and adaptation to cold stress stimuli in Bailinggu. Similarly, previous studies in other species have also found genes in these pathways, for example, during development in *Coprinopsis cinerea* ^40^ and other fungi^42^, and in response to cold and other abiotic stresses in *Tuber melanosporum* ^43^ and *P*. *ostreatus* ^44^. Taken together, these findings suggest that the above-mentioned pathways might be necessary for the growth of Bailinggu. Further research, via CRISPR/Cas9 or RNA interference systems, is needed to clarify the functions of these highly expressed genes so that the molecular mechanisms underlying Bailinggu mushroom development can be elucidated.

Blue light is a main environmental signal that triggers primordium and fruiting body formation including stipe elongation and cap formation, and influences the production in several edible mushrooms^45–53^. In the cultivation cycle of Bailinggu, we also found that blue light was essential for vegetative-to-fruiting transition and fruiting body growth. Although undifferentiated mycelia stages can proceed without light, the fruiting stages need a blue-light environment. We identified genes involved in blue-light response in Bailinggu, such as genes encoding white collar-1, cryptochrome, and photolyase. These results are consistent with previous studies of *C*. *cinerea*, *S*. *commune*, and *Lentinus edodes* ^54–56^. These studies demonstrated that white collar-1 and cryptochrome are blue-light photoreceptors, and that cryptochrome and photolyase families with the flavin adenine dinucleotide (FAD)-binding domain absorb blue light to perform different tasks. Therefore, we revealed candidate genes involved in Bailinggu’s light response that, in turn, might play a key role in mushroom photomorphogenesis, fruit body development, and yield formation. However, the photomorphogenesis process is extremely complex, requiring further study to understand the molecular mechanisms of these photomorphogenic genes.

We previously developed a few EST-SSR markers using mycelium-stage samples of Bailinggu^7^. In this study, we developed additional EST-SSR markers for genetic research of Bailinggu and verified their transferability in the *P*. *eryngii* species complex. We selected three different stage samples including mycelia, primodia and fruiting body of Bailinggu. We obtained three times more EST-SSR markers compared to our previous study^7^. The transferability rates of these EST-SSR markers in the two varieties of *P*. *eryngii* were relatively high (82% and 81%). The main reason for this high transferability might be that the three selected species were different, but too closely related, resulting in moderate cross-species conservation of SSR-containing genes. Teshom *et al*.^17^ also verified the higher transferability between closely related species. Therefore, we suggest that the newly developed EST-SSRs could be used for population identification and genetic studies within and among populations of the *P*. *eryngii* species complex populations. Additionally, the markers could be an important resource for further genetic studies in other related *Pleurotus* genus species.

Using Inter-Simple Sequence Repeat (ISSR) and Start Codon Targeted (SCoT) markers, previous studies showed the abundant genetic variability among the wild strains of Bailinggu from China^3^. In this study, using 26 EST-SSR markers, we also found that the relatively high level of genetic diversity and variability among the wild strains of Bailinggu from the northern part of Xinjiang Autonomous Region in China. We also observed that 45% of SSR alleles in the wild strains were lacking in the cultivated strains. These new or rare alleles from the wild strains will broaden the breeding gene pool of Bailinggu and support a long-term breeding program.

The taxonomical position of the wild Bailinggu is controversies in these years. Based on the phylogenetic analysis using four nuclear DNA fragments, Zhao *et al*.^1^ and He *et al*.^25^ suggested Bailinggu is a separate species of the *P*. *eryngii* species complex, namely *P*. *tuoliensis*. In this study, our findings based on the combined results of 26 EST-SSRs are in agreement with these reports that suggested Bailinggu be raised to the species level. According to the population structure analysis of the *P*. *eryngii* species complex populations, we observed that the introgression between the two varieties (Aweigu and Xingbaogu) of *P*. *eryngii* occurred at a higher level than between Bailinggu and the varieties. We speculate that this phenomenon may be due to differences in natural distributions and gene flow between Bailinggu and two varieties of *P*. *eryngii*. Population structure analysis results also revealed that the two varieties were similar to each other, whereas Bailinggu appeared more distinct and less similar to the two varieties. These verified genotype results closely matched the cultivated phenotype. Aweigu and Xingbaogu commonly have a pale brown cap, produce fruit without cold stimulation, and form columnar-shaped fruiting bodies^5^. In contrast, Bailinggu has a white cap, forms columnar- to palmate-shaped fruiting bodies. Moreover, most of Bailinggu strains need cold stimulation to fruit^5^. Therefore, we suggest that Bailinggu from China potentially originated from different ancestors than the two varieties of *P*. *eryngii* and evolved independently. The further verification is necessary via genome-level analysis, using whole genome sequencing of Bailinggu, followed by re-sequencing of the *P*. *eryngii* species complex population strains.

## Methods

### Strain source

Eighty-two strains of the *P*. *eryngii* species complex populations including Bailinggu, Aweigu, and Xingbaogu were used in this study. All 82 strains are maintained in the Engineering Research Center of the Chinese Ministry of Education for Edible and Medicinal Fungi, Jilin Agricultural University, China.

### Cultivation of Bailinggu

We selected four major developmental stage samples of cultivated strain CCMJ1077 of Bailinggu, which were provided by Hengdaxing mushroom cultivation factory in Beijing, China. The cultivation substrates using plastic bottles as containers contained 780 g medium including 5% corncob, 35% sawdust, 24% wheat bran, 10% maize powder, 4.5% soybean meal^7^. All samples used for RNA-seq were collected from the same batch of cultivated strain CCMJ1077. The factory firstly transferred the liquid culture from one fermentation tank to 2,000 cultivation bottles. These bottles were then grown at 25 °C for 60 days in the dark up to the physiological after-ripening stage (Stage I), which time the mycelia were fully colonized in each bottle. In the evening of the 60^th^ day, we randomly selected nine cultivation bottles from 2,000 bottles of stage I, and then took the mycelia from the top of the substrate of these nine bottles into the corresponding nine tubes and frozen at −80 °C for RNA extraction. Excepted for the nine cultivation bottles, the rest 1,991 cultivation bottles were then cultivated at −3 °C for 10 days in the dark (Stage II). In the evening of the 10^th^ day, we also randomly selected nine cultivation bottles from 1,991 bottles of stage II, and took the mycelia into the corresponding nine tubes.

For the rest 1,982 cultivation bottles, 2 mm of the colonized substrate and aerial mycelia were removed with a scratching machine. Then, these scratched bottles were cultivated under blue light up to primordial imitation (Stage III). We randomly selected nine cultivation bottles of stage III, and took the primordial into the corresponding nine tubes. We finally selected nine fruiting bodies (Stage IV) from the rest 1,973 cultivation bottles cultivating under blue light. All samples taken from nine selected bottles at the four developmental stages were frozen at −80 °C for RNA extraction.

### Library preparation and RNA-Seq

The total RNA was extracted from samples using TRIzol reagent (Life technologies, NY, USA) and evaluated for integrity and quality using an Agilent Technologies 2100 Bioanalyzer. For each stage, nine tubes of total RNA were extracted from the saved nine samples, and then equal amounts RNA of nine tubes were mixed into three new tubes. According to the manufacturer’s standard protocols, three cDNA libraries were constructed using these three mix tubes for each stage, for a total of 12 libraries. We then performed paired-end sequencing on all 12 libraries using the Illumina HiSeq 2500 platform by Biomarker Technologies (Beijing, China). For RNA-seq of each stage, these three cDNA libraries were used as three biological replicas samples. Sequencing data of these samples have been deposited in the National Center for Biotechnology Information (NCBI) database (SRP090421).

### *De novo* transcriptome assembly and homology search

Clean data were obtained by filtering and trimming the adapter, low-quality, and duplicate sequences from the raw reads. Then, *de novo* assembly was performed to obtain unigenes using Trinity’s standard protocol (http://trinityrnaseq.sf.net)^57^. These unigenes were annotated using the NCBI NR and Nt databases and the Interproscan, Swissprot, KOG (http://www.ncbi.nlm.nih.gov/COG), GO (http://wego.genomics.org.cn), and KEGG (http://www.genome.jp/kegg) databases^58–60^. Then, these unigenes were annotated using the genome sequences of *P*. *eryngii* (https://www.ncbi.nlm.nih.gov/genome/?term=Pleurotus+eryngii) and *P*. *ostreatus* (http://genome.jgi.doe.gov/PleosPC15_2/PleosPC15_2.home.html), and next clustered these annotated unigenes to the genome sequence of *P*. *eryngii*.

### Identification of differentially expressed genes

To identify the differentially expressed genes, genes expression values were first calculated by mapping clean read to unigenes database using RSEM^61, 62^, and a public software Samtools. Due to our samples with biological replicas, DEGs were then identified by R Bio-package DESeq. 2^63^. The p-value cut-off was set at 0.05 and log2 ratio at 1. Functional analyses of the DEGs were conducted using GO and KEGG pathways (http://www.kegg.jp/kegg/kegg1.html) (p-value ≤ 0.05 and |log_2_ ratio| ≥ 1)^58–60^.

### **S**SR mining, identification, and characterization

These unigenes were then used to identify and locate SSR loci with the MISA identification tool^7^. Primers for all SSR loci were designed using Primer 3 software (Whitehead Institute). Three hundred SSR primer-pairs were consistently synthesized by Beijing Dingguo Changsheng Biotechnology Co. Ltd. (Beijing, China), and then tested for polymorphism in *P*. *eryngii tuoliensis* and transferability in *P*. *eryngii* var. *eryngii* and var. *ferulae*. Genomic DNA was extracted from mycelia of these strains using the Plant DNA Mini Kit (Kangwei, Beijing, China); quality was identified by the NanoDrop 2000c spectrophotometer (Thermo Scientific, MA, USA). PCR amplification and polyacrylamide gel were performed as described in Fu *et al*.^7^.

### Genetic diversity and population structure analysis

Twenty-six polymorphic primer-pairs were selected from the initial 100 primer-pairs and used to explore the genetic diversity in 82 strains *P*. *eryngii* species complex populations. The forward primers of these 26 primer-pairs were 5′-labeled with 6-FAMTM fluorescent dye. The PCR procedure was the same as described above. The reaction volume was 20 μl, containing 10 μl 10 × PCR dream mix buffer (Thermo Scientific, MA, USA), 1 μl each labeled primer (2.0 μM), 1 μl DNA (20 ng/μl), and 8 μl ddH_2_O. PCR products were first electrophoresed on 2% agarose gels. A 50 bp DNA ladder (Thermo Scientific, MA, USA) was used as reference. Then, the successfully amplified PCR products were sequenced on capillary gel electrophoresis with an internal size standard (Genescan-500 ROX) using an ABI Prism 3730 DNA Analyzer (Applied Biosystems, CA, USA).

The sequence peak and fragment size were identified using GeneMapper software ver. 4.0 (Applied Biosystems). The genetic diversity parameters were calculated by GenAIEx (Ver. 6.502) and CERVE (Ver. 3.0.7) software. The genetic diversity analysis was carried out using the unweighted neighbor joining method by DARwin software (Ver. 5.0.145). The population structure was evaluated by STRUCTURE software (Version 2.3.4, Stanford University, Stanford, California, USA) and the STRUCTURE HARVESTER program (http://taylor0.biology.ucla.edu/structureHarvester/)^7, 64^.

## Electronic supplementary material

Supplementary Information

Supplementary Dataset S1

Supplementary Dataset S2

Supplementary Dataset S3

Supplementary Dataset S4

Supplementary Dataset S5

Supplementary Dataset S6

Supplementary Dataset S7

Supplementary Dataset S8
