# Supplementary material for: Comparative Transcriptome Analysis Identified Candidate Genes Related to Bailinggu Mushroom Formation and Genetic Markers for Genetic Analyses and Breeding
Source: Sci Rep. 2017 Aug 24;7:9266. doi: 10.1038/s41598-017-08049-z (PMC5571210; doi:10.1038/s41598-017-08049-z)
Supplement: Supplementary file 1 — Supplementary Information [file 41598_2017_8049_MOESM1_ESM.pdf]

Comparative Transcriptome Analysis Identified Candidate Genes Related to Mushroom Formation of Bailinggu and Genetic Markers for Genetic Analyses and Breeding

Yongping Fu[1, +], Yueting Dai[1, +], Chentao Yang[3], Peng Wei[4], Bing Song[1], Yang Yang[1], Lei Sun[1], Zhi-Wu Zhang[2*], Yu Li[1*]

1 Engineering Research Center of Chinese Ministry of Education for Edible and Medicinal Fungi, Jilin Agricultural University, Changchun, 130118, Jilin, China.

2 Department of Crop and Soil Sciences, Washington State University, Pullman, 99164, Washington, USA.

3 BGI-Shenzhen, Shenzhen 518083, China; China National GeneBank, Environmental Genomics, BGI-Shenzheng, Shenzhen, 518083, China;

4 Key Laboratory of Integrated Pest Management on Crop in Northwestern Oasis, Urumqi 830091 Xinjiang, China

[*] Corresponding author. Yu Li (yuli966@126.com).

[+] These authors contributed equally to this work.

Supplementary Figure S1-6

Supplementary Table S1-8

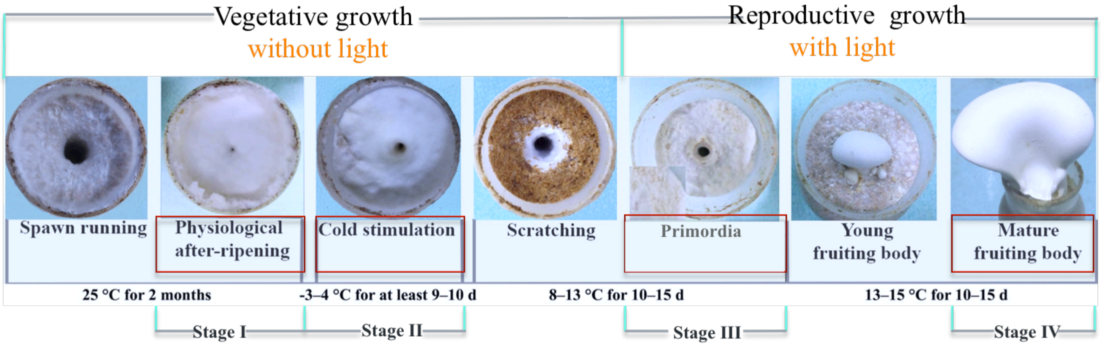

Supplementary Figure S1. Developmental stages of Bailinggu in year-round mushroom factory cultivation cycles. The vegetative (spawning, physiological ripening, cold stimulation) and fructification (primordia, fruiting body) phases are shown.

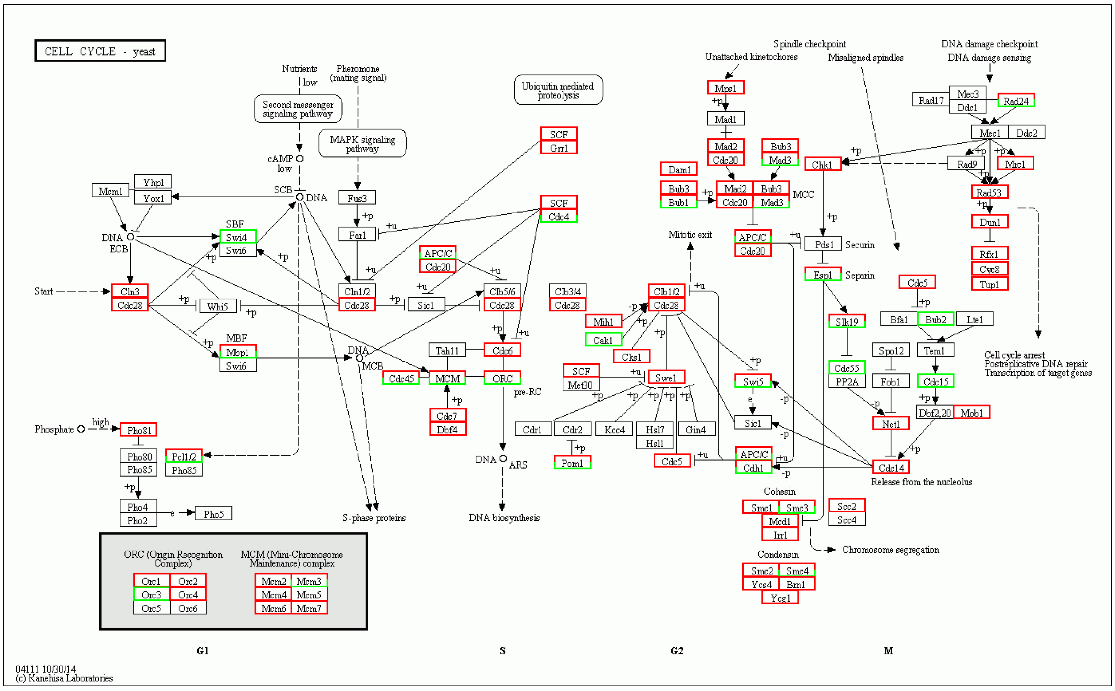

Supplementary Figure S2. The vegetative-to-reproductive transition stage related pathway-Cell cycle (http://www.kegg.jp/kegg/kegg1.html, derived from KEGG map04111) was identified by KEGG annotation [1-3]. The red and green boxes indicate that the up-regulation and down- regulation unigenes identified in the transcriptome of Bailinggu are annotated in the pathway, respectively.

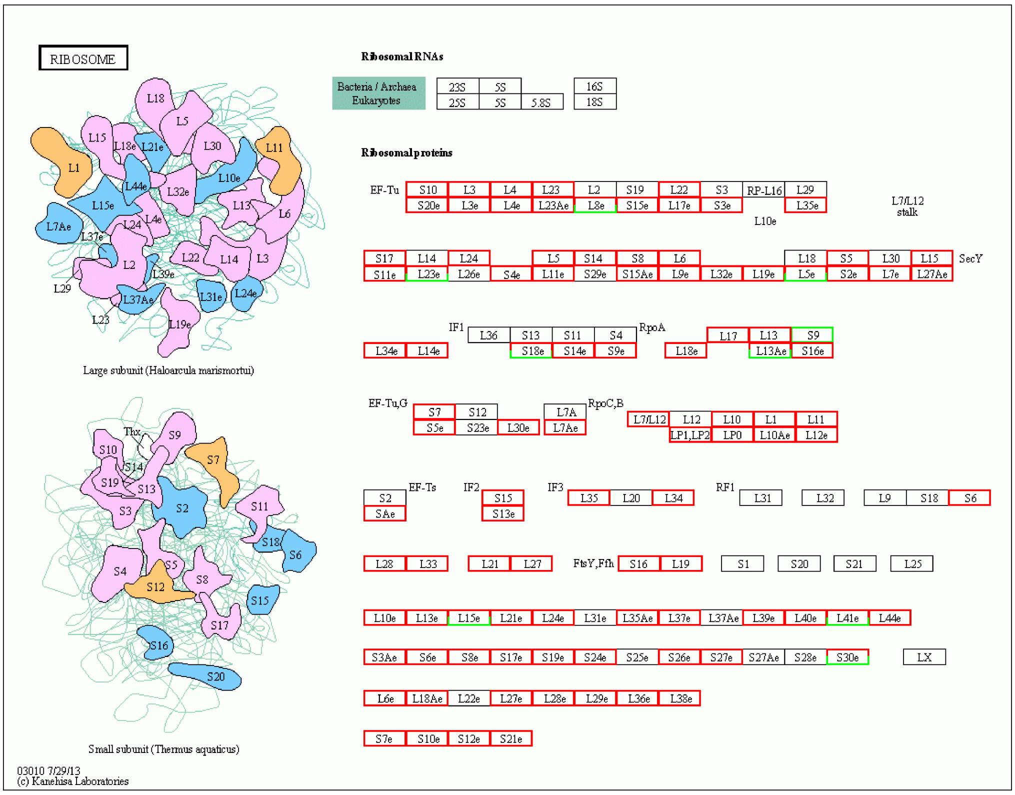

Supplementary Figure S3. The vegetative-to-reproductive transition stage related pathway-Ribosome (http://www.kegg.jp/kegg/kegg1.html, derived from KEGG map03010) was identified by KEGG annotation [1-3]. The red and green boxes indicate that the up-regulation and down-regulation unigenes identified in the transcriptome of Bailinggu are annotated in the pathway, respectively.

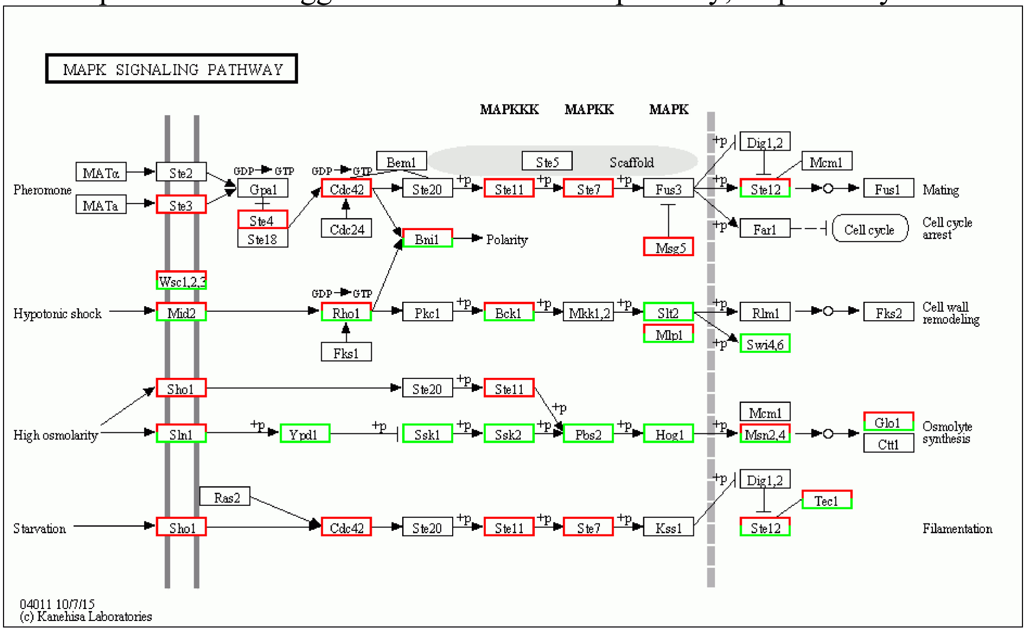

Supplementary Figure S4. The vegetative-to-reproductive transition stage related pathway-Mitogen-activated protein kinases (MAPK) signaling pathway (http://www.kegg.jp/kegg/kegg1.html, derived from KEGG map04011) was identified by KEGG annotation [1-3]. The red and green boxes indicate that the up-regulation and down-regulation unigenes identified in the transcriptome of Bailinggu are annotated in the pathway, respectively.

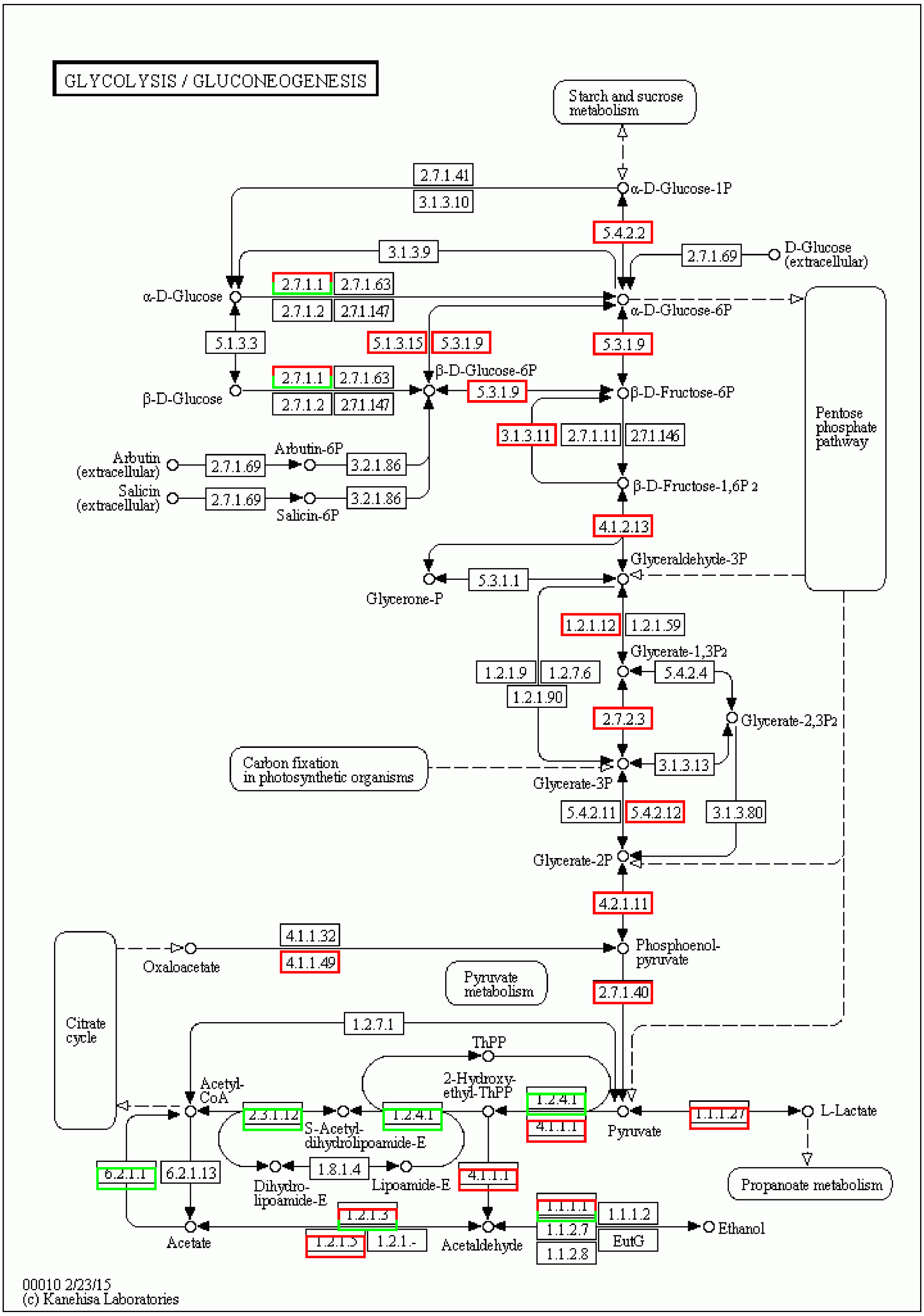

Supplementary Figure S5. The reproductive growth stage related pathway-Glycolysis (http://www.kegg.jp/kegg/kegg1.html, derived from KEGG map00010) was identified by KEGG annotation [1-3]. The red and green boxes indicate that the up-regulation and down- regulation unigenes identified in the transcriptome of Bailinggu are annotated in the pathways, respectively.

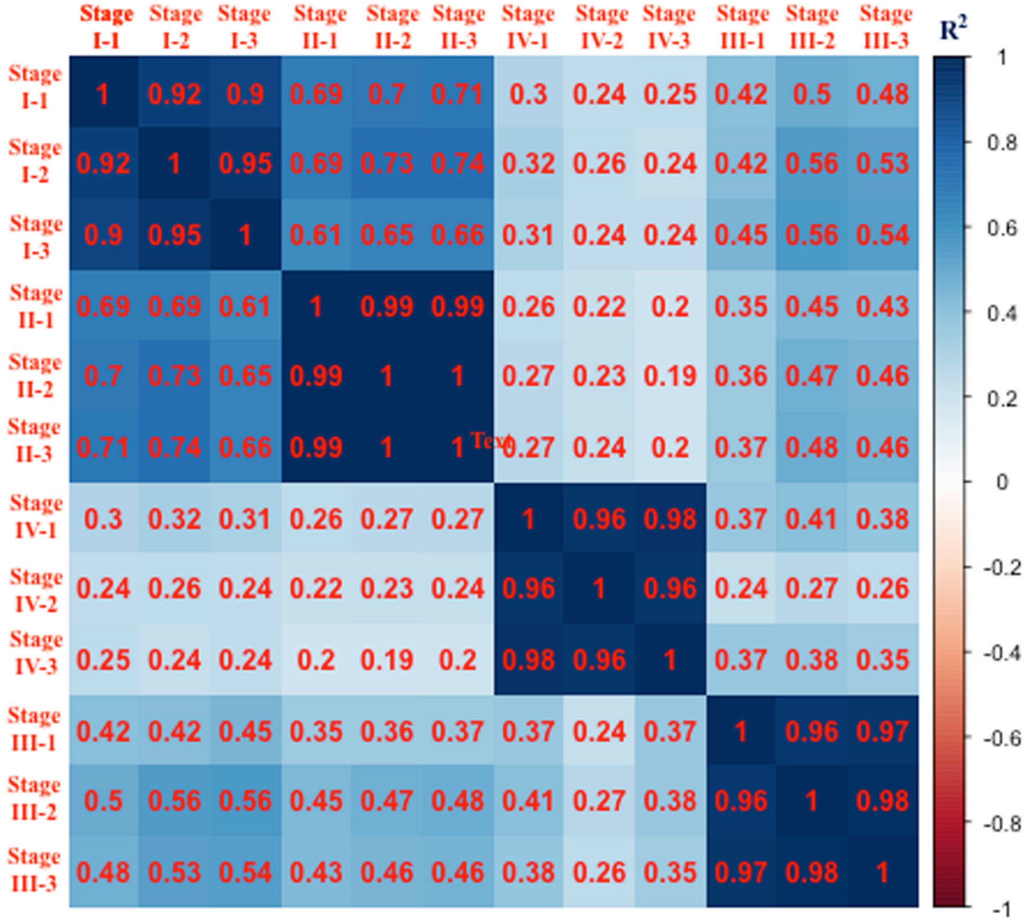

Supplementary Figure S6. Pearson correlation between samples using FPKM. The $\log_{10}$ (FPKM+1) of the samples are displayed along the x-axis and y-axis. R^2 represents the Pearson correlation coefficient squared.

Supplementary Table S1. Characteristics of the 100 novel EST-SSR markers used in this study.

Locus	Motif	Forward Primer (5'-3')	Reverse Primer (5'-3')	T_a (℃)	Polymorphism/Monomorphism/No product		
					Bailinggu	Aweigu	Xingbaogu
BlgSSR1	(CTC)6	GCATTCGAACT CTCCACCTC	GACTTCGATTC GCAAGAACC	60	P	P	P
BlgSSR2	(TCG)5	TGATCGTGGTC ATCTGGGTA	GTCCTATACCA GGCTGCTCG	60	P	P	P
BlgSSR3	(GAG)5	CGTCGGCATAT CGACTATCA	CTGGCTATGAC TCCCATCGT	60	P	P	P
BlgSSR4	(TA)7	ACTCGCACGA CACTTCACTG	CAAGGATGTG GAATGCAATG	60	P	M	P
BlgSSR5	(CAG)5	GCATACTCTCA GCCGTCCTC	TTAGAGAGTG GCGGTCGAAT	60	P	P	P
BlgSSR6	(TA)7	ATCTCACGTTG AGCAGGTCC	ACCTATCACGA GCCGCATAC	60	P	P	P
BlgSSR7	(CGC)5	TTCAGTTCAGC GAACCCTCT	TTAGCCTTCTC GACGGTGTT	60	P	P	P
BlgSSR8	(AT)7	TCCTCTATCTC GCCCATCAC	GGGACAGCCT ATTCGAGGAT	60	P	P	M
BlgSSR9	(TGA)5	TCTTGGTCGAA GAAAGCGAT	CTTTCCGAGAT GTACGCTCC	60	P	M	M
BlgSSR10	(TTG)5	CCAGAGGGCG TATAGTGGTC	AACGACCAGG ACACGATGAT	60	P	P	P
BlgSSR11	(CCA)5a(CAC)6ctcggtctcgatccctaccaaagctatctcgacgatcatcataatcatcgtaaccaccagggccgggaccaggg(CCT)6	TCCATAATTGT CATCTCCCCA	AAATAATTAC GACGGTGGGC	60	P	P	P
BlgSSR12	(CGA)6	ACGGCGATAC GAATGAAGTC	GGAAGACCAT TGCTGATCGT	60	P	P	P
BlgSSR13	(AT)9	GCCGCAAGAA ATATCCAAGA	TCAGTCAGGG GAAGTGAACC	60	P	M	P
BlgSSR14	(GTC)5gccgcc(GTCGGT)9	ATCTGTCTCGC GGTGATTCT	CTCGAGACCCT TCTTGACCA	60	P	P	P
BlgSSR15	(CCA)6	ACGTTGGAAA CTCCGACATC	GTCGATGGTAT CGGACGACT	60	P	P	P
BlgSSR16	(GCT)7gagcgggttgtccaaatagtccaccagaaggttgctgctgttgagaaggctgtccaaagagtcccccagtagtcccaga(CTG)5	ATGAACCGAA TCCACCAAAA	GGTAGTTTGTT CGGTCAGCC	60	P	P	P
BlgSSR17	(TGC)6	AGTGTTTGTTC TCGGGGTTG	TTCTGTTCCGT CAGCATCAC	60	P	P	P
BlgSSR18	(GCAAC)5	GTCCTGTCCAT CGATGTGTG	GTTGTCTCTCT TTCCGTCCG	60	P	P	P
BlgSSR19	(CAC)5	TGACCACCACC AATCGTAGA	CGTAACATCTG GGGAATGCT	60	M	P	P
BlgSSR20	(CCA)5	ATTGCACGATG ACGCTACAG	AAAATGGCAA GATCTGCACC	60	P	P	P
BlgSSR21	(GTG)6	ATTATCCTCCC TGCCTTCGT	CGTCTGAATGT CCACCCTTT	60	P	P	P
BlgSSR22	(CGT)6	TTCGACATCTT CTTCCCCAC	ACCATCCATAG GTGCTCGTC	60	P	P	P
BlgSSR23	(GGT)6	GAAGGTGCGA ATATAAGCGG	ACTACGAAGG CCGTCCCTAT	60	P	P	P
BlgSSR24	(GAG)14	ATCGGATTCGT TGTCGGTAG	CGGATTCCTCC TCTTCTTCC	60	P	P	P
BlgSSR25	(TG)6	TGCATCCTCCT CTGCTACCT	ATACAATCGCC TCCGATCAC	60	P	P	P
BlgSSR26	(ACA)5	AAGAGACCGC AAACGAAATG	CAAGGCGGGT GGTCTTAGTA	60	M	P	M

BlgSSR27	(CGT)5	TCTTTGGTTCC AGTCCCAAC	GAGCAGAAGA AGGGCAGCTA	60	P	M	P
BlgSSR28	(GAG)7	GAGCCTTCGA AACGTAGCAC	CCCACATATCA ACGTCATCG	60	P	M	M
BlgSSR29	(GGTA)5	CGGTACGCAG ATAGAGGGAG	ATCGTCTACCC AATGCCAAG	60	P	M	P
BlgSSR30	(CT)7ccgtttccttc tctctttctctttctcttt ctccg(TC)9	TTTCCCCGCTT TATTGTTTG	ATGGGGAATG GGGATAAGAG	60	P	N	N
BlgSSR31	(CTC)5	CAGCTCTGTCT TCTCCGTCC	GGTAAAGCAA CTCGAGCGAC	60	P	N	N
BlgSSR32	(GTG)6	GTAAATGTGG CGGAGGAAAG	CCACATGGAA TGCAGATGAG	60	M	P	M
BlgSSR33	(TGC)5	TCTCACGAGAT GGTGGTGAC	CTACGGTGCA GTCGTGTTGT	60	M	M	M
BlgSSR34	(GCG)5	CATCCAAATCC AAATCCCAC	ATCTGAGCCTG GACATCGAC	60	P	M	P
BlgSSR35	(CGC)5	AACATGTTCTC CAAGGCCAC	ATGCGTGCTAA CTGAACGTG	60	P	M	M
BlgSSR36	(TCG)6	TTCTTGGTGCT CGATATCCC	ACGGTGGCATT GAAGAAGAC	60	P	M	M
BlgSSR37	(CGA)7	ACAAAGGATA CGGCCAACTG	TTGCTTCGCTT GAAGAGTGA	60	P	M	P
BlgSSR38	(CGT)6	TTCGCTTCTTC GTTTTTCGT	GCTAAATGGA GGGTGCGTTA	60	P	N	N
BlgSSR39	(GCA)7	AGAAGAATGG CGGTCAGCTA	TGGTATAGTAT GGGTGGCGG	60	P	P	P
BlgSSR40	(CT)6	AAAGCTTAACT CACGCCCAG	TGGGACCATA GCTGGTTTTC	60	N	N	N
BlgSSR41	(GA)6	ATATGCGGAG GAGAGGGAGT	TGGCGTAGAG TTCTTCGGTT	60	P	P	P
BlgSSR42	(TC)7	CCACCCAAGTC ATTCGTTCT	AGAATCTCCCG CGTTTACCT	60	P	M	M
BlgSSR43	(TC)8	AGTAGTGTGC GTGCGAAGTG	GTTGATGCGGC TGGAATACT	60	P	N	N
BlgSSR44	(AT)9	CTCAATCGACA AGCAATGGA	ATGTGATGCTG CGACTCAAG	60	P	M	N
BlgSSR45	(AGC)6	GCTCGAAGAA AAGGCAACTG	CGAAAAGCCT AGATGCTCCA	60	M	N	N
BlgSSR46	(TGTA)6	CCGTTCCTTCT TTTGCTCTG	AGGTATACAA CCTGACGCGG	60	P	M	N
BlgSSR47	(GGC)7	CTTAACGTCGT CGTCCCATT	AACGTCGAGA CATACCGGAC	60	P	P	M
BlgSSR48	(ACCG)7	ACCCCTTACGG ACTAACGCT	CTGTTCTTGCT GTCCGTGAA	60	P	M	M
BlgSSR49	(AT)8	CCACCGATTCT TGGACTCAT	GAGTGTCCCG AAGTCTGAGC	60	P	M	M
BlgSSR50	(GGC)5	GGAGGTTGAC GTGAACGAAT	GCGAAGATTCT ACGAGTCCG	60	P	M	P
BlgSSR51	(CGA)6	CGGATCCGAA CCTAGTCAAA	GCCGGTATATT CGTCCTTGA	60	M	M	M
BlgSSR52	(CCG)5	CATCCTCGGGT TGATGATTT	CAGCGGACCTT ACTGAGGAG	60	N	N	N
BlgSSR53	(CG)8	CCCTCTGCAAA TCGTCTAGG	GAAATACGGG TATGCCAACG	60	P	N	N
BlgSSR54	(AT)7	CTCATTGCGCA CAACATTCT	CAATACCCTTT CTTGTCCGC	60	P	N	N
BlgSSR55	(GCG)7	AGGTGTGTTGG GAGGTGAAG	CGGCTCGTCAT TTATCCATT	60	P	P	P
BlgSSR56	(CG)6	TCGCCGTCGTC TAATTCTTC	AGAAAGAATG GGGAGGAGGA	60	N	N	N
BlgSSR57	(AT)8	CGCTTGGAGCT ACCTACTGC	AACGTAGATG ACGGGAGGTG	60	P	N	N
BlgSSR58	(ACC)5	GCAGATATTG ATGTCGCCCT	TGGAGTATAA GGTACGGCGG	60	P	P	N
BlgSSR59	(CTC)5	CCGAAAGTCC AAAGTCCAAA	ACCCATTGTCC GTTTTGTGT	60	P	N	N
BlgSSR60	(AAG)5	AAATGGGCTCT CGTGTCTTG	GCTTGGTGGAC AGAGGAGAG	60	P	P	M
BlgSSR61	(GAC)5	GACGAGCTCA TGAGTGGACA	CTCACTTCCGT AGTCGGCTC	60	M	M	M
BlgSSR62	(AT)6	TTGGTGGAGG AACTTTGAGG	GACGGATTCTT CGCACTCTC	60	P	P	M
BlgSSR63	(GAC)5	AGTTAGTAAA	GTGACCTTTGT	60	M	M	M

		CGCGCTCCGA	ACGCCGTCT				
BlgSSR64	(CG)6	AGTGGGAATA CGAACGAACG	CACGACACCTT ACCATGTGC	60	M	P	M
BlgSSR65	(GTC)5	CTAAATAACC GTTCGCCCAA	GTTCTGTGGAA GAAGCAGGC	60	M	M	M
BlgSSR66	(CT)6	TTCGCTCATCC TAAGGCACT	CGAGTGGGAA GAAGCGTAAG	60	M	P	P
BlgSSR67	(CTC)5	AAGGATCCCT GGACGTTTCT	ATCTCCGCTGT GGTCAATTC	60	M	M	M
BlgSSR68	(CAG)6	AATTCAAGGG AATTGGGGAC	GGTTGCTGTTG TGGTTGTTG	60	N	N	N
BlgSSR69	(CG)7	TCGAGTCTCCA AAGATGCCT	TGTGCCTCCTT AACCAGCTC	60	M	M	M
BlgSSR70	(TCC)5	TCGTGTCTCTA CGTTCGTGC	AGACGAGAAT GCGAACGACT	60	M	M	M
BlgSSR71	(CCA)5	ATTTCCATATC TTCCCCGCT	TCTTGAATCTG AGCACGCTG	60	P	M	M
BlgSSR72	(AG)7	CGGTGCCAGT GTTCCTTATT	TGATTCCTGCC CTTTCATTC	60	P	M	M
BlgSSR73	(CCGC)6	GCTAGTTTCTT GTCTGCCCG	ATTTAACGAGT GGGCCAGTG	60	P	M	M
BlgSSR74	(AATG)6	CATCTTAGCCG CTGGTTTTC	ATTCCGTAAGT TGGGCAGTG	60	P	P	M
BlgSSR75	(CGT)6	AGTGCCATATG ACCCAAAGC	CCCTATCGCAC AAAAAGCAT	60	M	N	P
BlgSSR76	(GAG)5	CGAGGAAGGC GAGACAATAC	GTCCTCGTCGT CCTCTTCAG	60	M	M	P
BlgSSR77	(TCA)6	ATGGTTACTTC GTGTTCCGC	CGTGCAGCAG ACTTTCCATA	60	P	P	P
BlgSSR78	(CGA)5	GAAAGCTCGC CATCAGACTC	AGAAGAAGCC CGAAGAGAGC	60	M	P	P
BlgSSR79	(CAA)6	CCACCACCTCG TTTCACTTT	AACTTGATCCA TGGGCTGAG	60	P	P	P
BlgSSR80	(TA)9	GCACCGACTAT TTGCCGTAT	TTAGGAACATC CGGTGAAGC	60	M	M	P
BlgSSR81	(AC)7	ACTGCGTCGTG GTGTACAAG	GTCGCTAAGTA TCGGTGGGA	60	P	N	N
BlgSSR82	(TCC)5	GTCCGCATCAT CATCTTCCT	CATAAGCCCG GTAGATCGAA	60	P	M	M
BlgSSR83	(TA)7	GCTAAACCTCT CATCGCCAG	TGGGCTGCAC AGACAATAAG	60	P	M	M
BlgSSR84	(GT)7	AAGGACACGC CAAAATGTTC	AAGTGGAACT GAAGCCATCG	60	P	N	N
BlgSSR85	(CGC)5	TTATTGAAGTT GCCTTGCCC	TTTCGGCTTCA TATCCTTCG	60	M	P	P
BlgSSR86	(CT)6	CTCCAAGAAG ATGTCGGAGC	GAGGACTTCCT CCTGCCTTC	60	P	N	N
BlgSSR87	(GGA)7	TGAAACTCGTC GTCTTGTGG	TTCACCAGGTC GTTCTCCTC	60	P	P	P
BlgSSR88	(GA)6	AGTCTCGAACC CAATCCCTT	CGTTGACCAA GACCCATTCT	60	P	P	P
BlgSSR89	(CG)6	GACCTCTGACC TCGTTCTGG	GAGGATGGAA CAGTGGAGGA	60	P	M	M
BlgSSR90	(TC)6	CTTATCGTCCC CGTTCTCAA	GTCCGGATTAC ACGGTGAAG	60	P	P	M
BlgSSR91	(TGC)5	CTCCCGGTATA CCTCGTTCA	ATGTCAATCGT CTAAGCCGC	60	P	M	P
BlgSSR92	(CT)7	GCATTTCACCA GTGTTGTGG	CGGTTCGTGTC TATGTGGTG	60	M	M	M
BlgSSR93	(TCA)5(TCG)7	AATACCGGTGT TCAAGCCAG	CCGCATCAAA CCCACTATCT	60	M	P	M
BlgSSR94	(CAG)5	GTTGAGAAGG AGCGTTCCAG	GGGAGAATGG GATTGGACTT	60	P	M	M
BlgSSR95	(CG)6	GTCACCCCCTC CATCCTATT	GGTACGTCATG TTCTGCACG	60	P	M	M
BlgSSR96	(CCT)5	CTCGTCAATAA CATCCGCCT	CCAAGAGGAG GACGAAGATG	60	P	M	M
BlgSSR97	(TA)7	CAATCTTGCTA CTCTCCGCC	AATCAACGAC TGTTGGAGGG	60	M	N	M
BlgSSR98	(GT)6(GC)6	GTACATCTGGG GAAGGGGAT	AGTGAGAAGT GGGGCTTCAA	60	P	P	P
BlgSSR99	(TCC)5	CGCATTGTCGG TCTCAGTAA	ATTAGGGCTG GTCGTTGTTG	60	M	P	P
BlgSSR100	(TCC)6	CCCCGTCCTCT AGTTCATCA	AATACGGGTC GTCAGATTCG	60	P	P	P

Supplementary Table S2. Detailed information for the three Pleurotus taxa populations.

Number	Strain name	Species	Strain types	Source
1	CCMJ964	*P. tuolesis* (Bailinggu)	Wild stain	Xinjiang, China
2	CCMJ968	*P. tuolesis* (Bailinggu)	Wild stain	Xinjiang, China
3	CCMJ969	*P. tuolesis* (Bailinggu)	Wild stain	Xinjiang, China
4	CCMJ973	*P. tuolesis* (Bailinggu)	Wild stain	Xinjiang, China
5	CCMJ979	*P. tuolesis* (Bailinggu)	Wild stain	Xinjiang, China
6	CCMJ980	*P. tuolesis* (Bailinggu)	Wild stain	Xinjiang, China
7	CCMJ981	*P. tuolesis* (Bailinggu)	Wild stain	Xinjiang, China
8	CCMJ994	*P. tuolesis* (Bailinggu)	Wild stain	Xinjiang, China
9	CCMJ997	*P. tuolesis* (Bailinggu)	Wild stain	Xinjiang, China
10	CCMJ1002	*P. tuolesis* (Bailinggu)	Wild stain	Xinjiang, China
11	CCMJ828	*P. tuolesis* (Bailinggu)	Cultivated strain	Jilin, China
12	CCMJ829	*P. tuolesis* (Bailinggu)	Wild stain	Xinjiang, China
13	CCMJ1123	*P. tuolesis* (Bailinggu)	Cultivated strain	Shandong, China
14	CCMJ1157	*P. tuolesis* (Bailinggu)	Cultivated strain	Heilongjiang, China
15	CCMJ1160	*P. tuolesis* (Bailinggu)	Wild stain	Xinjiang, China
16	CCMJ1161	*P. tuolesis* (Bailinggu)	Wild stain	Xinjiang, China
17	CCMJ1162	*P. tuolesis* (Bailinggu)	Wild stain	Xinjiang, China
18	CCMJ1165	*P. tuolesis* (Bailinggu)	Wild stain	Xinjiang, China
19	CCMJ1166	*P. tuolesis* (Bailinggu)	Wild stain	Xinjiang, China
20	CCMJ1170	*P. tuolesis* (Bailinggu)	Wild stain	Xinjiang, China
21	CCMJ1172	*P. tuolesis* (Bailinggu)	Wild stain	Xinjiang, China
22	CCMJ1173	*P. tuolesis* (Bailinggu)	Wild stain	Xinjiang, China
23	CCMJ1668	*P. tuolesis* (Bailinggu)	Cultivated strain	Beijing, China
24	CCMJ1669	*P. tuolesis* (Bailinggu)	Cultivated strain	Beijing, China
25	CCMJ1676	*P. tuolesis* (Bailinggu)	Cultivated strain	Xinjiang, China
26	CCMJ812	*P. tuolesis* (Bailinggu)	Cultivated strain	Xinjiang, China
27	CCMJ1148	*P. tuolesis* (Bailinggu)	Cultivated strain	Sichuan, China
28	CCMJ1667	*P. tuolesis* (Bailinggu)	Cultivated strain	Liaoning, China
29	CCMJ1171	*P. tuolesis* (Bailinggu)	Wild stain	Xinjiang, China
30	CCMJ1670	*P. tuolesis* (Bailinggu)	Cultivated strain	Beijing, China
31	CCMJ1077	*P. tuolesis* (Bailinggu)	Cultivated strain	Xinjiang, China
32	CCMJ814	*P. tuolesis* (Bailinggu)	Cultivated strain	Xinjiang, China
33	CCMJ983	*P. tuolesis* (Bailinggu)	Wild stain	Xinjiang, China
34	CCMJ816	*P. tuolesis* (Bailinggu)	Cultivated strain	Beijing, China
35	CCMJ1176	*P. tuolesis* (Bailinggu)	Wild stain	Xinjiang, China
36	CCMJ996	*P. tuolesis* (Bailinggu)	Wild stain	Xinjiang, China
37	CCMJ987	*P. tuolesis* (Bailinggu)	Wild stain	Xinjiang, China
38	CCMJ975	*P. tuolesis* (Bailinggu)	Wild stain	Xinjiang, China
39	CCMJ976	*P. tuolesis* (Bailinggu)	Wild stain	Xinjiang, China
40	CCMJ1097	*P. tuolesis* (Bailinggu)	Wild stain	Xinjiang, China
41	CCMJ999	*P. tuolesis* (Bailinggu)	Wild stain	Xinjiang, China
42	CCMJ1000	*P. tuolesis* (Bailinggu)	Wild stain	Xinjiang, China
43	CCMJ1001	*P. tuolesis* (Bailinggu)	Wild stain	Xinjiang, China
44	CCMJ1003	*P. tuolesis* (Bailinggu)	Wild stain	Xinjiang, China
45	CCMJ1119	*P. tuolesis* (Bailinggu)	Cultivated strain	Shanghai, China

46	CCMJ1118	*P. tuolesis* (Bailinggu)	Cultivated strain	Shanghai, China
47	CCMJ819	*P. tuolesis* (Bailinggu)	Cultivated strain	Beijing, China
48	CCMJ1163	*P. tuolesis* (Bailinggu)	Wild stain	Xinjiang, China
49	CCMJ1164	*P. tuolesis* (Bailinggu)	Wild stain	Xinjiang, China
50	CCMJ1167	*P. tuolesis* (Bailinggu)	Wild stain	Xinjiang, China
51	CCMJ1175	*P. tuolesis* (Bailinggu)	Wild stain	Xinjiang, China
52	CCMJ1044	*P. tuolesis* (Bailinggu)	Cultivated strain	Beijing,China
53	CCMJ1174	*P. eryngii* var. *ferulea* (Aweigu)	Wild stain	Xinjiang, China
54	CCMJ991	*P. eryngii* var. *ferulea* (Aweigu)	Wild stain	Xinjiang, China
55	CCMJ992	*P. eryngii* var. *ferulea* (Aweigu)	Wild stain	Xinjiang, China
56	CCMJ993	*P. eryngii* var. *ferulea* (Aweigu)	Wild stain	Xinjiang, China
57	CCMJ989	*P. eryngii* var. *ferulea* (Aweigu)	Wild stain	Xinjiang, China
58	CCMJ990	*P. eryngii* var. *ferulea* (Aweigu)	Wild stain	Xinjiang, China
59	CCMJ985	*P. eryngii* var. *ferulea* (Aweigu)	Wild stain	Xinjiang, China
60	CCMJ986	*P. eryngii* var. *ferulea* (Aweigu)	Wild stain	Xinjiang, China
61	CCMJ970	*P. eryngii* var. *ferulea* (Aweigu)	Wild stain	Xinjiang, China
62	CCMJ971	*P. eryngii* var. *ferulea* (Aweigu)	Wild stain	Xinjiang, China
63	CCMJ972	*P. eryngii* var. *ferulea* (Aweigu)	Wild stain	Xinjiang, China
64	CCMJ1150	*P. eryngii* var. *ferulea* (Aweigu)	Cultivated strain	Sichuan, China
65	CCMJ825	*P. eryngii* var. *ferulea* (Aweigu)	Cultivated strain	Shandong, China
66	CCMJ984	*P. eryngii* var. *ferulea* (Aweigu)	Wild stain	Xinjiang, China
67	CCMJ1717	*P. eryngii* var. *ferulea* (Aweigu)	Cultivated strain	Shandong, China
68	CCMJ1732	*P. eryngii* var.*eryngii* (Xingbaogu)	Cultivated strain	Jiangsu, China
69	CCMJ1066	*P. eryngii* var.*eryngii* (Xingbaogu)	Cultivated strain	Sichuan, China
70	CCMJ656	*P. eryngii* var.*eryngii* (Xingbaogu)	Cultivated strain	Fujian, China
71	CCMJ653	*P. eryngii* var.*eryngii* (Xingbaogu)	Cultivated strain	Fujian, China
72	CCMJ657	*P. eryngii* var.*eryngii* (Xingbaogu)	Cultivated strain	Fujian, China
73	CCMJ1072	*P. eryngii* var.*eryngii* (Xingbaogu)	Cultivated strain	Beijing, China
74	CCMJ1130	*P. eryngii* var.*eryngii* (Xingbaogu)	Cultivated strain	Fujian, China
75	CCMJ1683	*P. eryngii* var.*eryngii* (Xingbaogu)	Cultivated strain	Jilin, China
76	CCMJ1102	*P. eryngii* var.*eryngii* (Xingbaogu)	Cultivated strain	Shanghai, China
77	CCMJ1693	*P. eryngii* var.*eryngii* (Xingbaogu)	Cultivated strain	Liaoning, China
78	CCMJ1699	*P. eryngii* var.*eryngii* (Xingbaogu)	Cultivated strain	Shandong, China
79	CCMJ1707	*P. eryngii* var.*eryngii* (Xingbaogu)	Cultivated strain	Shandong, China
80	CCMJ645	*P. eryngii* var.*eryngii* (Xingbaogu)	Cultivated strain	Beijing, China
81	CCMJ1725	*P. eryngii* var.*eryngii* (Xingbaogu)	Cultivated strain	Shandong, China
82	CCMJ1731	*P. eryngii* var.*eryngii* (Xingbaogu)	Cultivated strain	Liaoning, China

Supplementary Table S3. Characteristics of the 26 polymorphic EST-SSR markers in the three Pleurotus taxa populations.

Locus	Samples	Na	Ne	Ho	He	I	PIC
BlgSSR1	82	7	2.6204	0.268	0.622	1.2293	0.561
BlgSSR2	82	7	3.284	0.256	0.7	1.4534	0.659
BlgSSR3	82	7	2.1455	0.232	0.537	1.148	0.505
BlgSSR4	82	6	3.9705	0.317	0.753	1.5705	0.716
BlgSSR5	82	4	2.5245	0.244	0.608	1.1106	0.553
BlgSSR6	82	10	3.1815	0.244	0.69	1.5109	0.651
BlgSSR7	82	10	3.6915	0.195	0.734	1.7037	0.703
BlgSSR8	82	6	2.2541	0.085	0.56	1.0367	0.49
BlgSSR9	82	2	1.8299	0.305	0.456	0.6459	0.351
BlgSSR10	82	9	3.5057	0.39	0.719	1.5501	0.677
BlgSSR11	82	10	4.7319	0.549	0.794	1.7942	0.761
BlgSSR12	82	10	3.5605	0.512	0.724	1.6051	0.686
BlgSSR13	82	10	6.1016	0.378	0.841	1.9542	0.816
BlgSSR14	82	6	3.9002	0.488	0.748	1.4601	0.7
BlgSSR15	82	13	4.4179	0.402	0.778	1.7817	0.741
BlgSSR16	82	11	4.2289	0.683	0.768	1.7514	0.737
BlgSSR17	82	8	2.5728	0.354	0.615	1.2892	0.571
BlgSSR18	82	10	3.6623	0.573	0.731	1.5917	0.687
BlgSSR19	82	11	2.2417	0.122	0.557	1.2786	0.53
BlgSSR20	82	6	2.8425	0.195	0.652	1.3241	0.609
BlgSSR21	82	6	2.6928	0.207	0.633	1.1458	0.555
BlgSSR22	82	3	2.8282	0.183	0.65	1.0677	0.572
BlgSSR23	82	12	2.6816	0.354	0.631	1.5508	0.611
BlgSSR24	82	10	4.712	0.683	0.793	1.8009	0.761
BlgSSR25	82	12	6.1688	0.476	0.843	2.1353	0.825
BlgSSR26	82	4	1.642	0.098	0.393	0.6829	0.339
Mean		8.077	3.384	0.338	0.674	1.429	0.629

Supplementary Table S4. Characteristics of the 26 polymorphic EST-SSR markers in the Xingbaogu population.

Locus	Samples	Na	Ne	Ho	He	I	PIC
BlgSSR1	15	3.000	1.402	0.333	0.287	0.534	0.26
BlgSSR2	15	2.000	1.142	0.000	0.124	0.245	0.117
BlgSSR3	15	5.000	2.778	0.467	0.640	1.249	0.594
BlgSSR4	15	4.000	2.473	0.600	0.596	1.073	0.539
BlgSSR5	15	2.000	1.301	0.267	0.231	0.393	0.204
BlgSSR6	15	4.000	2.113	0.200	0.527	0.899	0.445
BlgSSR7	15	4.000	3.383	0.067	0.704	1.289	0.651
BlgSSR8	15	1.000	1.000	0.000	0.000	0.000	0
BlgSSR9	15	1.000	1.000	0.000	0.000	0.000	0
BlgSSR10	15	2.000	1.069	0.067	0.064	0.146	0.062
BlgSSR11	15	3.000	2.632	0.933	0.620	1.030	0.548
BlgSSR12	15	3.000	2.663	0.800	0.624	1.028	0.545
BlgSSR13	15	3.000	2.486	0.800	0.598	0.995	0.526
BlgSSR14	15	2.000	1.471	0.400	0.320	0.500	0.269
BlgSSR15	15	5.000	2.778	0.133	0.640	1.203	0.581
BlgSSR16	15	3.000	2.228	0.867	0.551	0.882	0.456
BlgSSR17	15	6.000	3.846	1.000	0.740	1.541	0.703
BlgSSR18	15	3.000	2.419	0.800	0.587	0.970	0.509
BlgSSR19	15	6.000	1.807	0.333	0.447	0.996	0.43
BlgSSR20	15	3.000	1.613	0.467	0.380	0.683	0.342
BlgSSR21	15	2.000	1.867	0.067	0.464	0.657	0.357
BlgSSR22	15	2.000	1.385	0.067	0.278	0.451	0.239
BlgSSR23	15	3.000	2.406	0.467	0.584	0.948	0.495
BlgSSR24	15	3.000	2.866	0.933	0.651	1.074	0.576
BlgSSR25	15	4.000	2.528	0.733	0.604	1.058	0.529
BlgSSR26	15	1.000	1.000	0.000	0.000	0.000	0
**Mean**		3.077	2.064	0.415	0.433	0.763	0.384

Supplementary Table S5. Characteristics of the 26 polymorphic EST-SSR markers in the Aweigu population.

Locus	Samples	Na	Ne	Ho	He	I	PIC
BlgSSR1	15	2.000	1.385	0.200	0.278	0.451	0.239
BlgSSR2	15	2.000	1.142	0.133	0.124	0.245	0.117
BlgSSR3	15	2.000	1.642	0.000	0.391	0.580	0.315
BlgSSR4	15	1.000	1.000	0.000	0.000	0.000	0
BlgSSR5	15	3.000	1.402	0.333	0.287	0.534	0.26
BlgSSR6	15	4.000	2.239	0.067	0.553	1.057	0.515
BlgSSR7	15	4.000	3.285	0.067	0.696	1.282	0.644
BlgSSR8	15	3.000	2.018	0.400	0.504	0.831	0.428
BlgSSR9	15	1.000	1.000	0.000	0.000	0.000	0
BlgSSR10	15	5.000	3.543	0.667	0.718	1.385	0.67
BlgSSR11	15	2.000	1.965	0.200	0.491	0.684	0.371
BlgSSR12	15	7.000	3.237	0.533	0.691	1.484	0.659
BlgSSR13	15	1.000	1.000	0.000	0.000	0.000	0
BlgSSR14	15	3.000	1.495	0.267	0.331	0.591	0.294
BlgSSR15	15	9.000	3.689	0.733	0.729	1.682	0.704
BlgSSR16	15	6.000	4.286	0.733	0.767	1.580	0.731
BlgSSR17	15	2.000	1.142	0.133	0.124	0.245	0.117
BlgSSR18	15	2.000	1.142	0.133	0.124	0.245	0.117
BlgSSR19	15	6.000	3.879	0.333	0.742	1.522	0.702
BlgSSR20	15	3.000	1.226	0.067	0.184	0.389	0.175
BlgSSR21	15	3.000	1.145	0.133	0.127	0.291	0.123
BlgSSR22	15	2.000	1.069	0.067	0.064	0.146	0.062
BlgSSR23	15	5.000	3.409	0.267	0.707	1.315	0.649
BlgSSR24	15	7.000	3.383	0.400	0.704	1.474	0.661
BlgSSR25	15	5.000	2.446	0.667	0.591	1.058	0.507
BlgSSR26	15	4.000	2.184	0.533	0.542	1.000	0.495
Mean		3.615	2.129	0.272	0.403	0.772	0.3675

Supplementary Table S6. The differentially expressed unigenes (DEGs) between four different developmental stages.

Supplementary Table S7. FPKM data of all samples.

Supplementary Table S8. All GeneExpression FPKM data.

References

1. Kanehisa, Furumichi, M., Tanabe, M., Sato, Y., and Morishima, K. KEGG: new perspectives on genomes, pathways, diseases and drugs. *Nucleic Acids Res.* **45**, D353-D361 (2017).
2. Kanehisa, M., Sato, Y., Kawashima, M., Furumichi, M., and Tanabe, M. KEGG as a reference resource for gene and protein annotation. *Nucleic Acids Res.* **44**, D457-D462 (2016).
3. Kanehisa, M. and Goto, S. KEGG: Kyoto Encyclopedia of Genes and Genomes. *Nucleic Acids Res.* **28**, 27-30 (2000).
